# Exploring the influence of silicon oxide microchips shape on cellular uptake using imaging flow cytometry

**DOI:** 10.1007/s00604-024-06631-7

**Published:** 2024-08-21

**Authors:** Gordon Bruce, Saman Bagherpour, Marta Duch, José Antonio Plaza, Snow Stolnik, Lluïsa Pérez-García

**Affiliations:** 1 Division of Advanced Materials and Healthcare Technologies, School of Pharmacy, University of Nottingham, Nottingham, NG7 2 UK; 2 Departament de Farmacologia, Facultat de Farmàcia I Ciències de L’Alimentació, Toxicologia I Química Terapèutica, Universitat de Barcelona, Av. Joan XXIII 27-31, 08028 Barcelona, Spain; 3 Institut de Nanociència i Nanotecnologia (IN2UB), Universitat de Barcelona, 08028 Barcelona, Spain; 4 Instituto de Microelectrónica de Barcelona IMB-CNM (CSIC), Campus UAB, Cerdanyola del Vallès, 08193 Barcelona, Spain; 5 Division of Regenerative Medicine and Cellular Therapies, School of Pharmacy, University of Nottingham, Nottingham, NG7 2 UK

**Keywords:** Cellular uptake, Functionalization, Imaging flow cytometry, Fluorescence label, Microfabrication, Silicon oxide microchips

## Abstract

Nano- and micro-carriers of therapeutic molecules offer numerous advantages for drug delivery, and the shape of these particles plays a vital role in their biodistribution and their interaction with cells. However, analysing how microparticles are taken up by cells presents methodological challenges. Qualitative methods like microscopy provide detailed imaging but are time-consuming, whereas quantitative methods such as flow cytometry enable high-throughput analysis but struggle to differentiate between internalised and surface-bound particles. Instead, imaging flow cytometry combines the best of both worlds, offering high-resolution imaging with the efficiency of flow cytometry, allowing for quantitative analysis at the single-cell level. This study focuses on fluorescently labelled silicon oxide microchips of various morphologies but related surface areas and volumes: rectangular cuboids and apex-truncated square pyramid microchips fabricated using photolithography techniques, offering a reliable basis for comparison with the more commonly studied spherical particles. Imaging flow cytometry was utilised to evaluate the effect of particle shape on cellular uptake using RAW 264.7 cells and revealed phagocytosis of particles with all shapes. Increasing the particle dose enhanced the uptake, while macrophage stimulation had minimal effect. Using a ratio particle:cell of 10:1 cuboids and spheres showed an uptake rate of approximately 50%, in terms of the percentage of cells with internalised particles, and the average number of particles taken up per cell ranging from about 1–1.5 particle/cell for all the different shapes. This study indicates how differently shaped micro-carriers offer insights into particle uptake variations, demonstrating the potential of non-spherical micro-carriers for precise drug delivery applications.

**Graphical Abstract:**

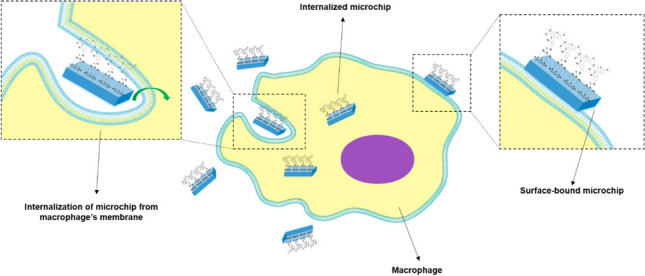

**Supplementary Information:**

The online version contains supplementary material available at 10.1007/s00604-024-06631-7.

## Introduction

Nano- and microscale particle carriers, designed to transport therapeutic molecules, provide numerous benefits for biomedical applications, including drug delivery, biosensing, targeted cancer therapies, gene editing, and regenerative medicine [1]. Recently, the amount of research in this area has risen drastically; although there still remain many hurdles to increasing the number of approved pharmaceutical products [2], some associated with their cellular uptake, influenced by some features of the particles such as particle size, surface chemistry, charge, and mechanical properties. Development of the research in this area provides opportunities to rationally design drug delivery systems based on the desired therapeutic outcome.

Particle shape is also an important determinant in the biodistribution of particles after administration to the body, and particular attention has been paid to the movement of micron-sized particles of different shapes in flow [3]. The hydrodynamics of non-spherical microparticles in blood often result in their margination towards the blood vessel walls, leading researchers to explore their use in vascular targeting [4]. Decuzzi et al. [5] have demonstrated differences in biodistribution between spheres, disks, cylinders, and hemispherical particles, opening another avenue for consideration in the targeting of different areas of the body using different particle shapes.

Studies of the uptake of silicon oxide particles of different shapes have primarily been focussed on respirable silica dusts in order to understand how the particle morphology affects toxicity in macrophages [6–8]. Recently, an increased number of studies have begun to examine silicon oxide particle morphology as a tool for enhancing control over drug delivery, for example by modulating uptake by cells [9–12].

It is difficult to make clear comparisons between these studies because the effects of particle shape are difficult to study in isolation and because experimental conditions tend to not coincide. Differences in particle size, surface chemistry, and cell type are ever present and have been shown to play important roles in particle internalisation [13–15]. Additionally, cellular association is often used as a proxy for internalisation. Moreover, the majority of studies examining the effect of silicon oxide particle shape utilise particles that are in the nano size range [16–20], whereas there are fewer studies that look at silicon oxide particles in the micron size range (> 1 μm).

The methodology of analysing particle internalisation is a key aspect to be considered. There are numerous methods available to assess the internalisation of particles into cells in vitro, which can be divided into qualitative or semiquantitative methods such as confocal/fluorescence/light microscopy and scanning electron microscopy and quantitative (flow cytometry, plate reader methods, inductively coupled mass spectrometry (ICP-MS)) [21]. Qualitative methods rely on high-resolution imaging to allow visualisation of particle internalisation events and are useful in providing descriptive information about the sample. However, often these methods are time-consuming and expensive which therefore leads to a reduction in the number of samples that can be assessed, thus decreasing the reliability of the quantitative information that can be obtained. Furthermore, due to the selection of which cells are imaged by the researcher, these methods can be subject to unconscious sampling bias that can reduce the validity of the obtained results [22].

On the other hand, quantitative methods are able to assess high numbers of cells in order to give highly reliable information about the sample but lack the sensitivity in discriminating between internalised and surface-bound particles [23]. For example, assessment of cells by flow cytometry or plate reader methods will give information about the proportion of cells that are associated with fluorescent particles, but not the location of particles with respect to the cells. Further methodological steps can be used, for example by quenching extracellular fluorescence or by staining surface-bound particles with a secondary fluorophore to discriminate between cells with internal and cells with surface-bound particles. However, these methods are very system specific, requiring efficient quenching/labelling, and therefore are not suitable as a general method.

Imaging flow cytometry (IFC) is a recently developed method that aims to overcome some of these limitations by combining the spatial resolution of microscopy with the high-throughput nature of flow cytometry. IFC generates quantitative image data for every event present, such as cells, cell clusters, and debris, which enables the detailed analysis of individual cells within diverse populations, contributing to our knowledge of cellular heterogeneity [24]. IFC has been used by a number of groups to assess the internalisation of nanoparticles, and it requires particle fluorescent labelling [25, 26].

Our group has expertise in the functionalization of hexahedral-shaped silicon-based microchips, fabricated by silicon-based technologies, for intracellular pH sensing [27] which showed that the chemical integrity and functionality of immobilised compounds (proteins, antibodies, or fluorophores) remain after conjugation with these types of microchips. Moreover, we have demonstrated the versatility of particle size and shapes that can be fabricated using photolithography [28, 29] followed by exhibiting the low polydispersity micro-fabricated particles in the aqueous media [30, 31]. However, the effect of silicon oxide microparticle shape is yet to be investigated.

In this work, silicon oxide microchips (or microparticles) of rectangular cuboid and apex-truncated square pyramid morphologies, which are called cuboids and pyramids, fabricated using photolithography, are compared with silicon oxide microspheres acquired from a commercial supplier to assess their interactions with RAW 264.7 macrophages. All microchips offer different morphologies but similar surface areas and volumes initially. Two siloxane linkers of different length were used for the functionalization of the microchips with fluorescent labels, and the interaction with RAW 264.7 macrophages of the functionalised particles was assessed using IFC.

## Experimental

The Materials and General Methods sections are included in the supporting information.

### Silicon oxide microchips fabricated by silicon-based technologies for the production of chips

For silicon oxide microchips preparation, the wet oxidation method was applied to grow the silicon oxide layer with a thickness of 1 μm on the surface of silicon and the plasma-enhanced chemical vapour deposition (PECVD) to deposited a silicon oxide layer with a thickness of 3 µm. The main process parameters of the 1-µm thermal oxide layer are as follows: 1100 °C and growing time 2 h 25 min. Meanwhile, the parameters for the 3-µm PECVD silicon oxide layer are as follows: 400 °C, plasma power 400 W, pressure 9 torr, gases (TEOS 500 SCCM and O2 SCCM), and deposition time 6 min 15 s. Then, on the silicon oxide layer, spinning a positive photoresist with a thickness of 1.2 μm was performed. Afterward, UV light through the photomask was applied for defining the shape of particles, as well as the lateral nominal dimensions (3 × 3 µm^2^). After that, the C_2_F_6_ and CHF_3_ mixture were used for dry etching the silicon oxide layer, followed by the photoresist stripping. Then, anchors were defined underneath particles by employing quasi-isotropic reactive ion etching (RIE). Finally, silicon oxidation (1100 °C and 5 h 15 min) was applied to convert the silicon pillars in silicon oxide pillars [27], and this annealing also makes more comparable the PECVD silicon oxide to the thermal grown one.

### Covalent labelling and removal of silicon oxide microchips anchored on silicon wafers

Silicon wafers possessing silicon oxide microchips attached to the surface by supporting anchors were cut using a diamond scribe into ~ 5 × 5 mm pieces. Pieces of wafer were transferred to a 10-mL glass vial (four wafer pieces per vial) with the silicon oxide particle pattern facing up. Acidic piranha solution (1 mL) was prepared by adding H_2_O_2_ (35%, 300 μL) dropwise to H_2_SO_4_ (98%, 700 μL), and this solution transferred into the vial containing the wafer pieces. This solution was freshly prepared each time to avoid loss of activity. After 1 h, the acidic piranha solution was removed, and the surfaces rinsed with Milli-Q water (3 × 1 mL), dried under a nitrogen stream, and added to a fresh 10-mL glass vial.

Basic piranha solution was prepared by adding NH_4_OH (20%, 150 μL) to Milli-Q water (700 μL), and to this mixture, H_2_O_2_ (35%, 150 μL) was added dropwise. Basic piranha solution was transferred to the vial containing the wafer pieces for 30 min. This solution was freshly prepared each time to avoid loss of activity. The basic piranha solution was removed, and the wafer pieces washed with Milli-Q water (3 × 1 mL) and dried under a stream of nitrogen before being transferred to a fresh 10-mL glass vial.

Freshly activated wafer pieces were placed in a 10-mL glass vial with the silicon oxide particle facing up and submerged in siloxane solution: either 2% v/v APTMS solution in acetone (1 mL) for 30 min or 0.1% v/v AUTES solution in EtOH (1 mL) for 2 h. Siloxane solutions were removed, and the wafer pieces washed with 3 × 1 mL of the corresponding solvent (acetone for APTMS-treated wafer pieces, EtOH for AUTES treated wafer pieces) before being dried with a nitrogen air stream and placed in a fresh 10-mL glass vial. RBITC solution in EtOH (1 mL) was then added to the wafer pieces which were protected from light and left overnight. For experiments comparing the covalent labelling methods, a 200-μM solution of RBITC in EtOH (1 mL) was used, and for all subsequent experiments, 30-μM RBITC solution in EtOH (1 mL) was used. Alternatively, BDP 630 NHS ester (30 μM) in Na_2_HPO_4_/NaH_2_PO_4_ buffer pH 8 (700 μL) was added to the wafer pieces, protected from light and left overnight. Wafer pieces were washed with 3 × 1 mL EtOH (RBITC-labelled wafer pieces) or Milli-Q water (BDP 630-labelled wafer pieces) and dried with a stream of nitrogen before removal of particles by peeling.

For the removal of silicon oxide particles from surface, a drop of Fluoromount™ aqueous mounting medium was placed onto each of the wafer pieces and spread using a micropipette tip to ensure complete coverage. This was then left to air-dry (~ 20 min). After drying, the mounting medium was peeled away from the wafer piece surface using tweezers and placed in a 1-mL microcentrifuge tube [27]. Milli-Q water (1 mL) was added to the microcentrifuge tube and sonicated to aid dissolution of the mounting medium. After complete dissolution had occurred, the microcentrifuge tube was centrifuged for 10 min at 5700 RPM (6175 G), resulting in a pellet forming. The supernatant was removed, and the cycle of centrifugation and washing with Milli-Q water (1 mL) was repeated two times. Particles were stored in EtOH (700 μL) at 4 °C.

### Covalent labelling of spherical silicon oxide microchips in suspension

A total of 1 mg (20 μL) silicon oxide spheres as received (stock concentration 5% w/v) were transferred to a 1.5-mL microcentrifuge tube and centrifuged at 5700 RPM (6175 G) for 10 min and the supernatant removed. Particles were suspended in acidic piranha solution (700 μL) for 1 h with shaking to prevent particles from falling out of suspension. Particles were centrifuged at 5700 rpm (6175 G) for 10 min and the acidic piranha solution removed. Particles were washed by resuspension in 700-μL Milli-Q water and then centrifuged 5700 rpm (6175 G) for 10 min. This washing process was repeated three times and then the supernatant removed. Particles were suspended in basic piranha solution (700 μL) and left shaking for 30 min. Particles were centrifuged at 5700 rpm (6175 G) for 10 min and the basic piranha solution removed. Particles were washed using the same resuspension in 700-μL Milli-Q water and centrifugation steps used to wash the particles after removal of the acidic piranha solution.

Freshly activated silicon oxide particles were suspended in 0.1% v/v AUTES in EtOH (700 μL) and left to shake for 2 h. Particles were centrifuged at 5700 RPM (6175 G) for 10 min and the silane solution removed. Particles were washed by resuspension in 700-μL EtOH and centrifugation at 5700 RPM (6175 G) for 10 min before removal of the supernatant. This washing process was repeated three times and then the supernatant removed. Particles were suspended in RBITC (30 μM) in EtOH (700 μL) or BDP 630 NHS ester (30 μM) in Na_2_HPO_4_/NaH_2_PO_4_ buffer pH 8 (700 μL) and left on a shaker overnight while protected from light. Particles were washed with EtOH 3 × 700 μL (RBITC-labelled particles) or Milli-Q water 3 × 700 μL (BDP 630-labelled particles) by repeated suspension and centrifugation as above. Particles were stored in 700-μL EtOH at 4 °C.

### Determination of trypan blue quenching efficiency

Measurement of median fluorescence intensity (MFI) of particles was measured by imaging flow cytometry using a 50-μL suspension containing RBITC-labelled particles in 1% HEPES in HBSS. MFI was measured with and without the addition of trypan blue solution (0.4%, 10 μL) immediately before sample analysis. Flow cytometer settings were kept the same for each sample, and details can be found in the flow cytometry section.

### Qualitative particle uptake of silicon oxide microchips by RAW 264.7 macrophages assessed by confocal microscopy

BDP 630-labelled particles were centrifuged at 5700 RPM (6175 G) for 10 min. From this point onwards, all work took place inside a sterile cell culture hood to maintain the sterility of the particles. Stock particle suspensions were prepared by removal of the EtOH and the addition of 1-mL buffer comprising 1% HEPES in HBSS. A volume of each particle stock solution containing 1,500,000 particles was transferred into fresh microcentrifuge tubes and the volume made up to 1 mL with fresh 1% HEPES in HBSS buffer.

RAW 264.7 cells grown to 60–80% confluency were harvested by scraping and counted by the protocol described in the supporting information. A total of 300,000 cells per well in 1-mL culture media were seeded onto collagen-coated glass coverslips inside clear polystyrene 6-well plates and incubated at 37 °C, 5% CO_2_, and 95% humidity overnight to allow cell attachment. Culture media was then removed, and the cells washed with 1-mL pre-warmed (37 °C) PBS. The PBS was removed by aspiration, and 1 mL per well pre-warmed particle suspensions each containing 1,500,000 particles added to each well. Cells were kept in an incubator for 4 h after which time the particle suspensions were removed by aspiration and the cells washed with 3 × 1 mL pre-warmed PBS. Cells were fixed by the addition of 1 mL per well of 4% formaldehyde in PBS for 15 min and then washed with 3 × 1 mL PBS. Permeabilization of cells was achieved by adding 1 mL per well 0.1% Triton X-100 in PBS for 3 min. Cells were washed with PBS (3 × 1 mL) and the PBS removed. The immunofluorescence staining and preparation of confocal slides are explained in the supporting information.

### Quantitative uptake of silicon oxide microchips by RAW 264.7 cells assessed by flow cytometry

RBITC-modified particles were prepared according to the same procedure explained in the “Preparation of fixed cells for flow cytometry: imaging flow cytometry (IFC) and trypan blue quenching (TBQ) method” section. RAW 264.7 cells were also harvested once they reach 60–80% confluency by scraping according to the described protocol in the supporting information. A total of 100,000 cells in culture media were seeded per well into a 12-well plate and incubated overnight at 37 °C, 5% CO_2_, and 95% humidity. In experiments where RAW 264.7 cells were stimulated with LPS, culture media was used without 1% penicillin/streptomycin and was supplemented with 100-ng per mL LPS. Culture media was removed by aspiration and the cells washed with 1-mL pre-warmed (37 °C) PBS. PBS was removed and 1 mL pre-warmed particle suspensions applied at ratios of 5 or 10 particles per cell. Cells were incubated for 4 h after which time the particle suspensions were removed and the cells washed with warm HBSS (3 × 1 mL). Cells were prepared for flow cytometry as either fixed or live cells depending on the analysis method.

### Preparation of fixed cells for flow cytometry: imaging flow cytometry (IFC) and trypan blue quenching (TBQ) method

For both IFC method and TBQ, HBSS was removed from each well by aspiration and 250-μL Accutase added to each well, and the cells were placed in an incubator for 5 min to detach cells. A total of 250-μL HBSS was then added to each well and the total volume of each well transferred to fresh microcentrifuge tubes. Samples were centrifuged at 250 G for 5 min and the supernatants removed.

In the IFC experiment, cells were suspended in 250-μL 4% formaldehyde in PBS for 20 min before being centrifuged for 5 min (250 G) and the supernatant removed. Fixed cells were suspended in 50-μL HBSS and stored at 4 °C until analysis by flow cytometry. Samples were always analysed within 1 week of sample preparation. Regarding the TBQ method, cells were suspended in 50-μL HBSS and kept on ice until analysis by flow cytometry. All samples were analysed within 2 h of sample preparation. Immediately before analysis of each sample 10 μL 0.4%, trypan blue solution was added to quench surface-bound particle fluorescence.

## Results and discussion

### Characterisation of fabricated microchips

**Scheme 1:**
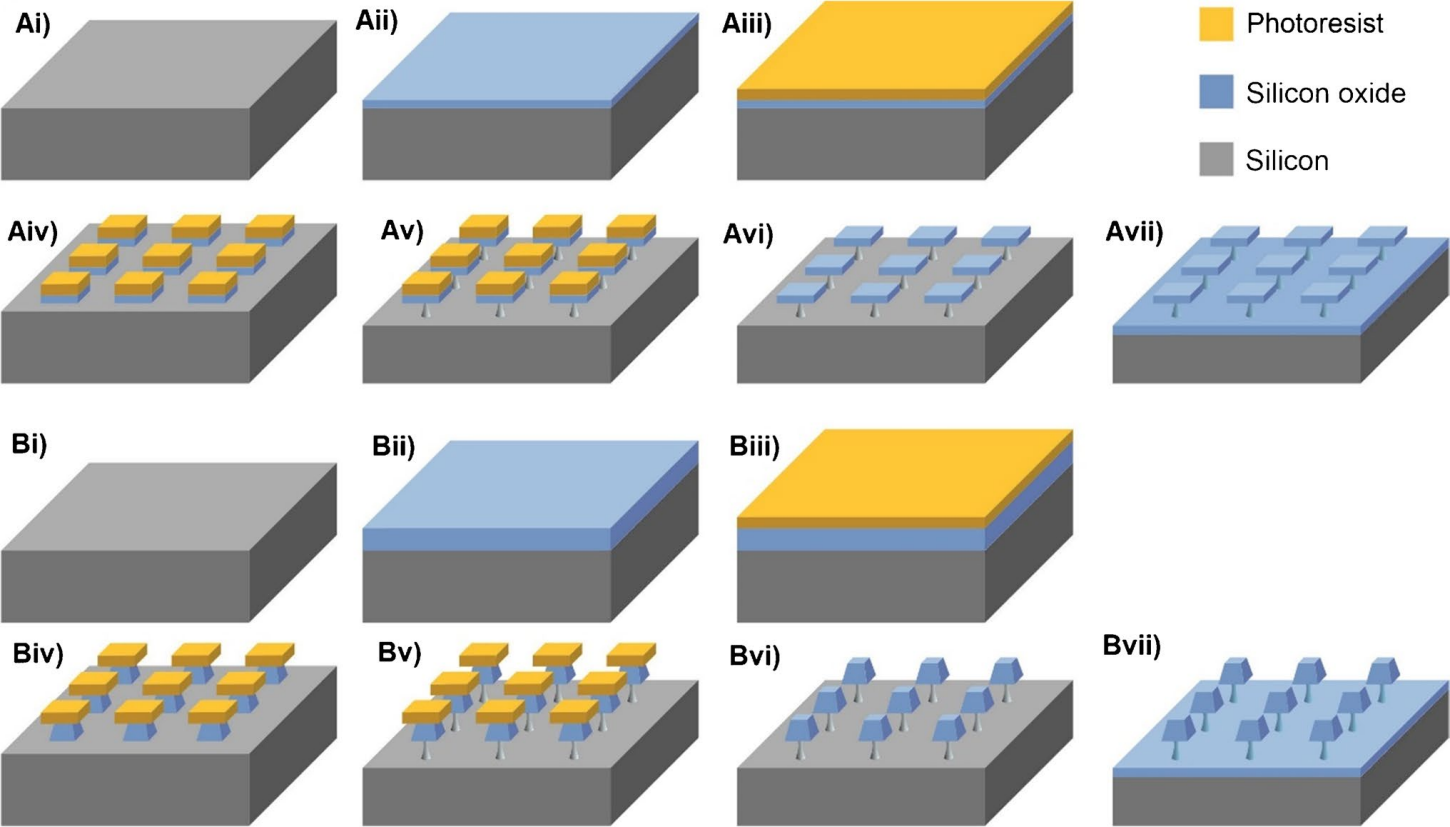
Fabrication of **A** cuboids and **B** pyramids silicon oxide microchips. Ai, Bi Silicon substrate, Aii, Bii growing or depositing silicon oxide layer on the surface of silicon, Aiii, Biii spinning a positive photoresist on the silicon oxide layer, Aiv, Biv defining the dimensions of microchips by the utilisation of UV light through the photomask and a dry etching (RIE) of the silicon oxide layer, Av, Bv defining anchors by RIE process of the silicon substrate, Avi, Bvi photoresist removal, and Avii, Bvii silicon oxidation to convert the silicon pillars in silicon oxide pillars

Silicon oxide microchips anchored on to silicon wafers were microfabricated using photolithography (Scheme 1). The method involves growing a thermal layer of silicon oxide on top of a silicon wafer (nominal 1-µm thick for the cuboids) and a PECVD deposition (nominal 3-µm thick for the pyramids), and a photomask is then used to selectively etch the particles that have a morphology which is defined by the photomask and the thickness of the original silicon oxide layer. After fabrication, particles remain anchored to the silicon wafer by a thin silicon support. This silicon support is thermally oxidised to allow single material microchips. This oxidation also anneals the PECVD oxide (3-µm pyramids) to make it more similar to the grown silicon oxide (1-µm cuboids). The anchors are posteriorly broken to release the particles into suspension.

**Fig. 1:**
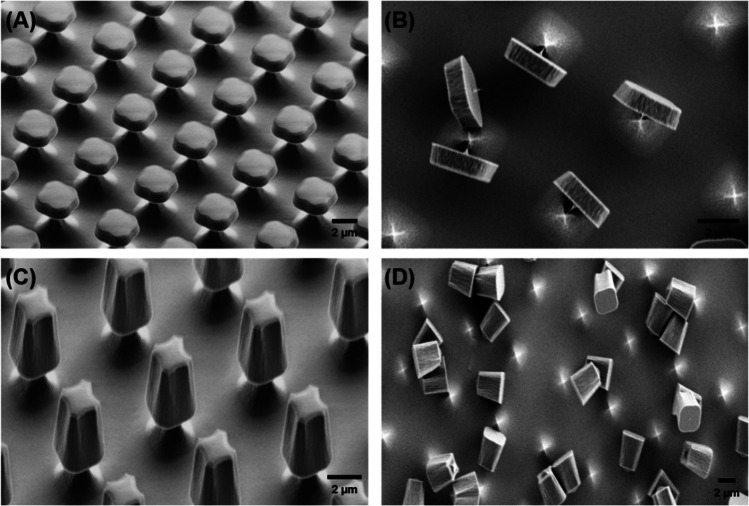
SEM images of fabricated silicon oxide microchips. **A** Cuboids, **B** zoom detail of cuboid microchips, **C** pyramids, and **D** zoom detail of pyramid microchips

SEM images of microfabricated silicon oxide microchips show highly uniform particle dimensions for both cuboids and pyramids (Fig. 1A and C). To ensure that all microchips are of the same size and feature precision, several key factors were controlled rigorously during the photolithography process, including alignment precision, uniform exposure of UV lights, and equipment calibration. In addition, environmental factors like temperature, humidity, and vibration were tightly controlled in the fabrication facility (cleanroom) to prevent any variations in the process. Figure 1B and D shows cuboid and pyramid microchips, respectively, removed from their silicon support, and a ca. 200-nm piece of the support pillar remains attached to the particle. Uniformity of particle size and shape is essential to ensure that any effects of particle shape on cellular uptake in later experiments are not caused by polydispersity. Particle dimensions were measured using ImageJ®, and the results are displayed in Table S1 in the supporting information.

Both cuboids and pyramids have a wider base than at the top of the particles, and some rounding can be observed on the particle edges due to the limit of our photolithographic and etching processes, which is particularly evident in the pyramids. Surface area and volume of the microchips are related, although lateral dimensions are dissimilar, so are their morphology. For the calculation of particle volume and surface area, this curvature was not taken into account, and flat edges assumed. Silicon oxide spheres, acquired from a commercial supplier, had a diameter of 3 μm according to the manufacturer’s analysis.

### Labelled silicon oxide microchips anchored on silicon wafers characterisation

**Fig. 2:**
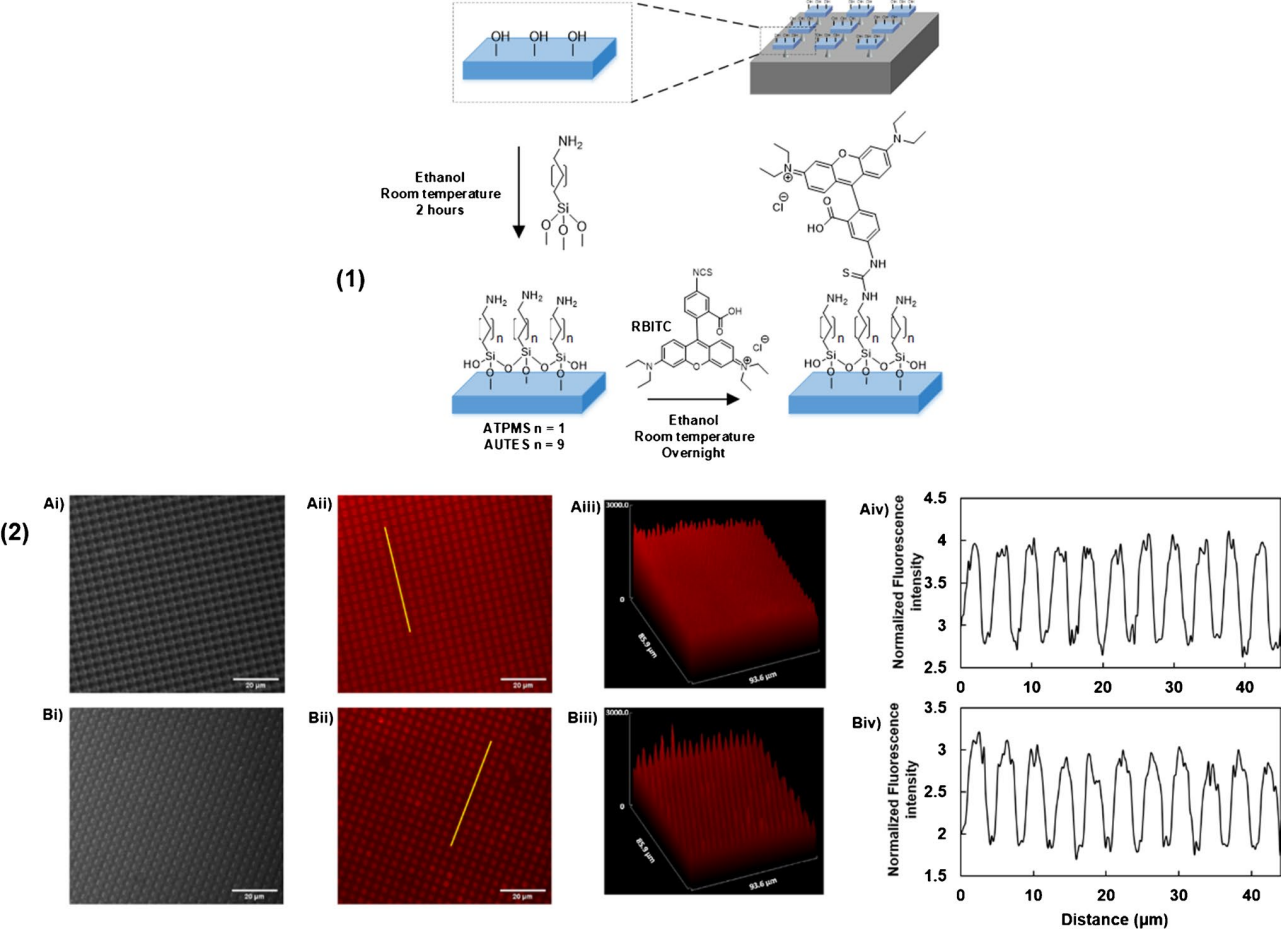
1 Functionalization sequence for labelling of activated silicon oxide microchip surfaces with silane linkers APTMS/AUTES and RBITC. **2** Fluorescence of covalently labelled particles. A APTMS and RBITC and B AUTES and RBITC. i Brightfield, ii fluorescence, iii fluorescence surface plots, and iv fluorescence intensity profiles. Yellow lines on Aii/Bii represent the location from which fluorescence line plots Aiv/Biv were taken

Covalent labelling methods have been used by a vast number of groups in order to label silica nanoparticles with fluorescent molecules or other molecules of interest [32], and from the wide variety of silanes available, 3-aminopropyltrimethoxysilane (APTMS) and 11-aminoundecyltriethoxysilane (AUTES) were chosen (Fig. S1), in order to explore the influence of the spacer link in the functionalization process. APTMS and AUTES are both amine-terminated silanes, which offer stable terminal groups that can be coupled with amine reactive fluorophores such as rhodamine B isothiocyanate (RBITC) (Fig. 2 (1)).

Successful activation of the surface, using acidic and basic piranha treatment, was confirmed by measuring water contact angle; activated surfaces had a water contact angle < 10° indicating hydrophilic nature due to hydroxyl groups present on the surface. After activation, siloxanes were deposited on particles attached to wafers and their water contact angles measured. The results are shown in Table S2. The increase in contact angle from hydrophilic (< 10°) to hydrophobic (> 40°) is consistent with the presence of hydrocarbon chains of the silanes and thus indicates successful deposition of each of the silanes. APTMS and AUTES displayed comparable water contact angles to those reported previously in the literature [33]. APTMS exhibits lower contact angles (ca. 38°) than AUTES (ca. 61°) due to the shorter length of its hydrocarbon chains [34].

RBITC was added to APTMS- and AUTES-modified surfaces as shown in Fig. 2 (1). Fluorescence microscopy images of functionalized chips on surfaces showed that each of the silanes had been successfully immobilised onto the particle surface, which had subsequently been labelled (Fig. 2 (2)). In each case, plots of the normalised fluorescence intensity of the surfaces indicate the uniformity of the labelling on the surface of the particles, which was very consistent and exhibits surface homogeneity with the average normalised fluorescence intensity of 3.98 ± 0.07 (a.u.) measured from analysing 500 microchips. This is important for cell studies as an uneven distribution of fluorophore on the particle surface will cause a difference in interaction between the particle and cell and thus interfere with the results. AUTES was chosen as the linker for subsequent experiments because it has been demonstrated that linkers with longer carbon chains form stable monolayers that are more resistant to hydrolysis than shorter chains [33], which can be relevant to insure the stability of the labelling.

Prior to the uptake experiments, it was necessary to determine the stability of the fluorophores on the labelled particles, to ensure no fluorophore would release in buffer conditions at 37 °C. Wafer pieces possessing particles labelled covalently using AUTES-RBITC were incubated in PBS at 37 °C for 2 h. The fluorescence intensity of the particles was assessed before and after incubation using fluorescence microscopy (Fig. S2 in the supporting information). Median fluorescence intensity values were calculated by imaging 5 areas of each wafer piece and measuring the fluorescence intensity of 500 particles per area. The results show that no significant decrease in fluorescence intensity was observed with covalently labelled AUTES-RBITC particles.

### Characterisation of fluorescently labelled microchips in suspension

#### Measurement of median fluorescence intensity (MFI) and surface coverage of fluorophore

Silicon oxide spheres, cuboids, and pyramids were fluorescently labelled using AUTES and RBITC. For the spheres, this process was performed in suspension, whereas cuboids and pyramids were fluorescently labelled while still attached to wafers by a supporting anchor followed by peeling away from the wafer by using a mounting medium as shown in Scheme S1 in the supporting information. All resulting labelled particles in suspension were imaged using fluorescence microscopy (Fig. S3 in the supporting information) revealing their successful labelling. Distinct particle shapes can be identified in the fluorescence images which correspond to the brightfield images indicating that RBITC is evenly distributed across the surface of the particles, and that the labelling was not affected by the release of the particles from the wafer. The normalised fluorescence intensity measured from fluorescent microscopy images for spheres, released cuboids, and released pyramids in the suspension was 3.67 ± 0.06 (a.u.), 3.57 ± 0.07 (a.u.), and 3.84 ± 0.08 (a.u.) for ~ 200 particles for each type, indicating the homogenous labelling according to standard deviations related to each type of particles.

In order to compare the degree of fluorescent labelling of each particle type, imaging flow cytometry was also used to measure the median fluorescence intensity (MFI) (*n* > 1000 particles of each shape). The obtained values and fluorescence intensity histograms are included in Fig. S4 (supporting information). The values show that each particle type has similar MFI values indicating a similar degree of labelling. To confirm this, the percentage of RBITC-labelled surface was estimated. This was done by comparing the MFI values of each particle type with a calibration curve of particles (3-μm spheres) that had known percentages of labelled RBITC (Fig. S5 in supporting information), and the results show that all particles had lower than 2% of surface labelled with RBITC.

It is important to have a similar and reduced amount of RBITC on the surface of the particles because rhodamine B is a relatively hydrophobic molecule (logP 1.95) and could promote uptake into cells [35]. Juliano et al. found that the uptake of dendrimers into HeLa cells was enhanced by their conjugation with Oregon Green, although the exact mechanism was not deduced [36]. Additionally, it has been shown that rhodamine B interacts with type BI scavenger receptors and so could enhance uptake via specific receptor interactions [37]. Importantly, by having a low percentage of surface amines labelled with rhodamine, these effects on interaction with the cells will be reduced. In a study looking at the required amount of particle surface functionalisation to enhance cellular uptake, it was found that at least 4% of the surface functional groups (maleimide) needed to be covered with wheat germ agglutinin (WGA) in order to significantly increase uptake of PEG-PLA nanoparticles into Calu-3 cells in comparison with non-functionalised particles [38].

##### Measurement of trypan blue quenching efficiency

To distinguish internalised particles from surface-bound particles, we used a protocol based on fluorescence quenching of surface-bound particles. In order for this method to be effective, the degree of fluorescence quenching must be known to ensure that surface-bound particles are significantly less fluorescent than internalised particles. To calculate this, the MFI of each particle type was measured before and after quenching with trypan blue (*n* > 1000 particles). The results are also displayed in Fig. S4 (1), showing that in each case the MFI was decreased after trypan blue quenching by over 90%, allowing distinction of internalised and surface-bound particles.

### Microchip interaction with RAW 264.7 cells

#### Qualitative determination of microchips uptake

**Fig. 3:**
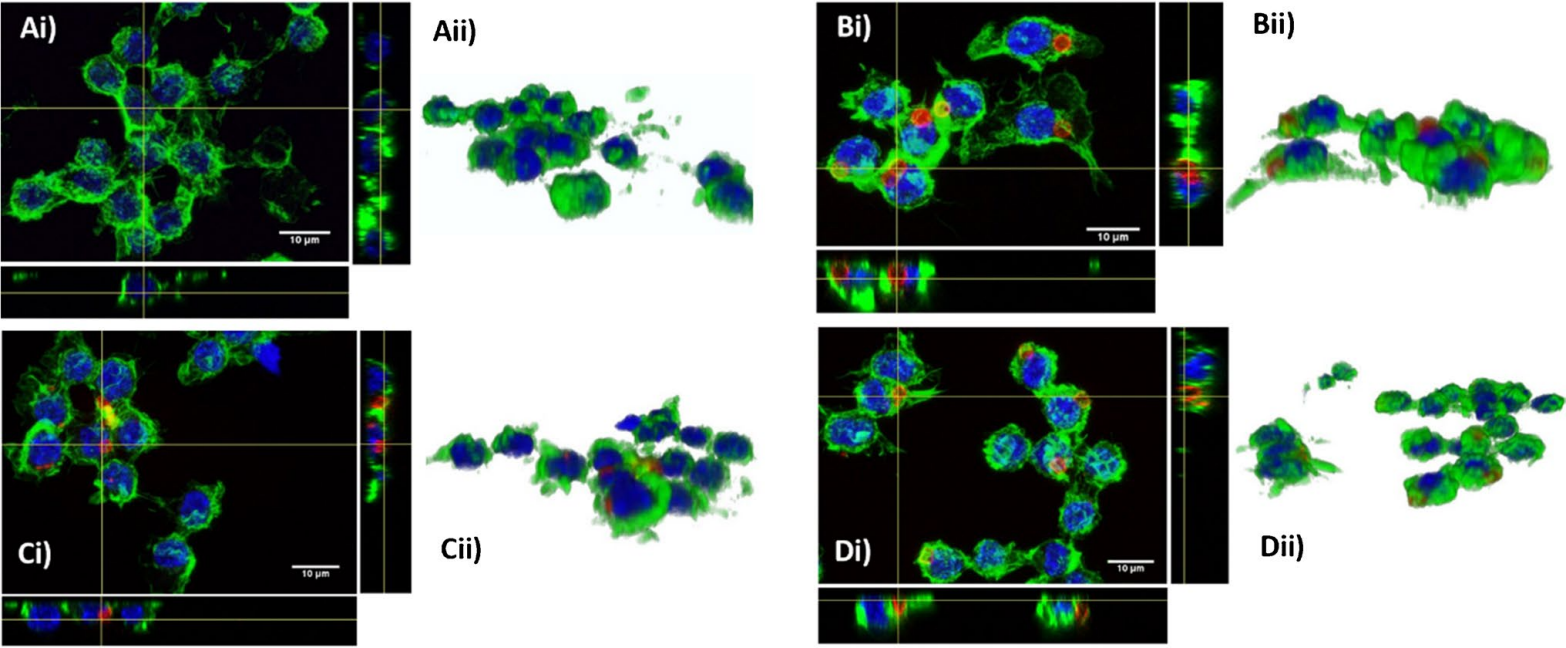
LSCM images of RAW 264.7 cells with internalised microchip shapes. i Z projections and orthogonal views, ii 3D reconstructions of cells treated with **A** 1% HEPES in HBSS only, **B** spheres, **C** cuboids, and **D** pyramids. Nuclear staining (blue), actin staining (green), and particles (red)

To examine if each of the differently shaped microchips could be internalised by RAW 264.7 macrophages, laser scanning confocal microscopy (LSCM) and immunostaining were used. Macrophages were incubated with fluorescently labelled particles of each shape for 4 h before being fixed and stained. BDP 630 was used in place of RBITC to fluorescently label the particles for this qualitative examination of particle uptake. Representative Z-projections are displayed along with their orthogonal views and 3D representations in Fig. 3. Cell nuclei, stained with DAPI, are shown in blue, actin filaments stained with iFluor 488 phalloidin are shown in green, and particles are displayed in red. Actin staining with phalloidin shows part of the cell cytoskeleton, either with cells appearing rounded or in some cases spread out with pseudopodia seen protruding from cells. Particles appear as hollow shapes in the orthogonal views (Fig. 3Bi, Bii, Ci, Cii, Di), because they are labelled only on their surface and so no fluorescence is seen inside the particles. In comparison with control cells that had not received any particle treatment (Fig. 3A), macrophages, which had been treated with particles, displayed similar morphologies and nuclear shapes. The actin cytoskeleton staining (green) can be used to determine if the particles have been internalised. In the orthogonal views, particles found within the green boundary of the cytoskeleton can be considered to have been internalised. These images show that each of the particle shapes had been internalised by RAW 264.7 macrophages. Particles were typically found in the perinuclear region of the cell which is in line with the established literature which details that upon phagocytosis, particles are contained within the phagosome which is transported towards the nucleus fusing with lysosomes to become a phagolysosome [39].

### Intracellular localisation of spheres, cuboids, and pyramids

**Fig. 4:**
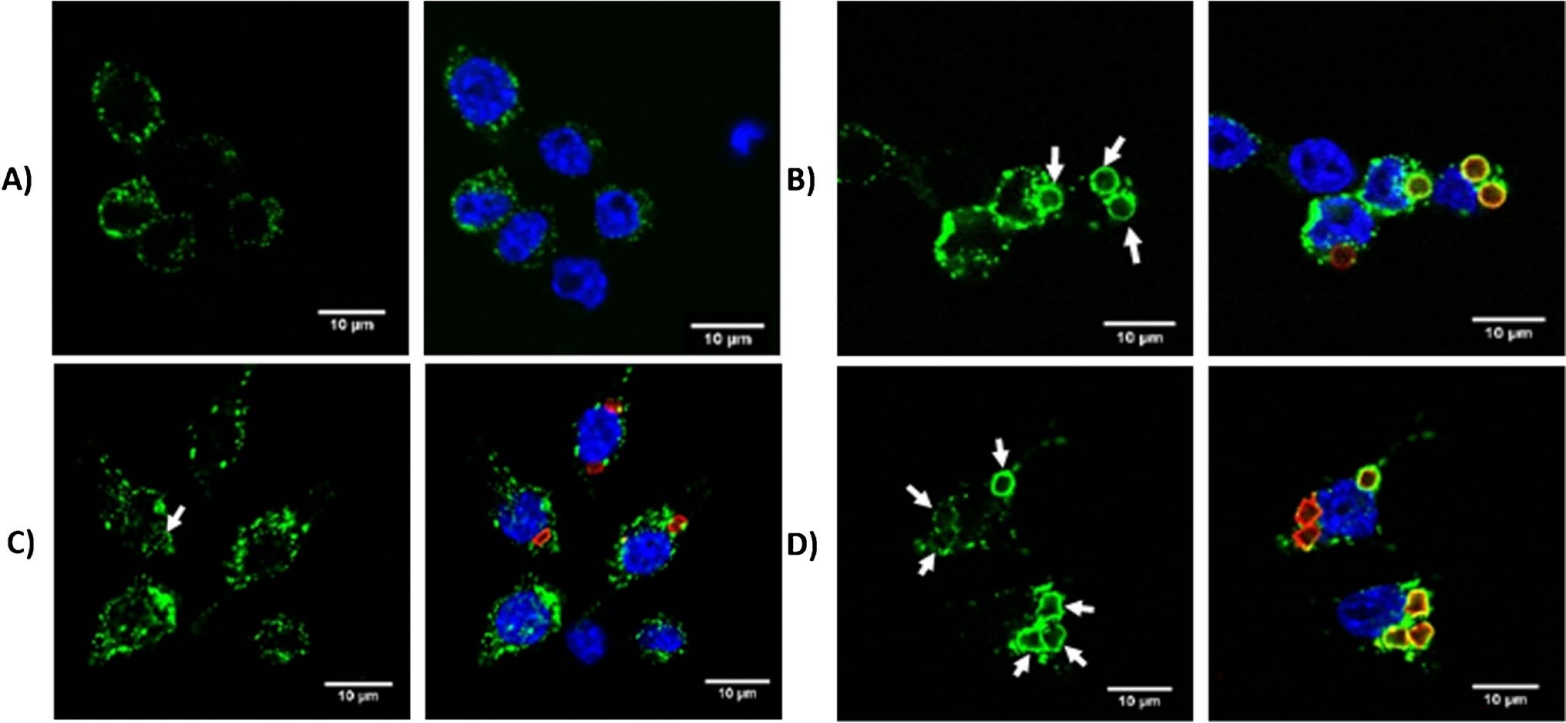
Intracellular localisation of particles after phagocytosis. Cells treated with **A** 1% HEPES in HBSS only, **B** spheres, **C** cuboids, and **D** pyramids. DAPI (blue), LAMP-1 (green), particles (red). White arrows indicate LAMP-1 colocalisation with particles

Immunostaining was used to determine the intracellular localisation of the particles after internalisation had taken place. Cells were incubated with particles for 4 h and then fixed and permeabilised. Cell nuclei were stained blue with DAPI, and lysosomal-associated membrane protein 1 (LAMP-1) antibody stain (green) was applied to stain lysosomes/late endosomes. Images of stained cells with each particle shape are displayed in Fig. 4 with particles displayed in red. In untreated cells (Fig. 4A), lysosomes/late endosomes were stained and appeared as small green dots inside the cell surrounding the nucleus. Lysosomes/late endosomes were not stained in all cells, and this could be because not all cells contained lysosomes/late endosomes or more likely due to a lack of staining, perhaps resulting from a lack of permeabilization. In cells to which particles had been applied, lysosomes/late endosomes could also be seen as small green dots throughout the cell. However, in cells that had internalised particles, a green ring was often observed surrounding the particle. This indicates that LAMP-1 was present in the membrane surrounding the particle. Upon maturation of the phagosome, lysosomes and endosomes fuse with the phagosomal membrane to deposit their contents. A ‘kiss-and-run’ mechanism has been proposed as a major mechanism for this, where complete fusion of the lysosomal/endosomal and phagosomal membranes is prevented by fission after some transfer of luminal contents has taken place [40]. Proteins such as LAMP-1 are left behind in the late phagosome/phagolysosome, thereby indicating that fusion has taken place [41]. The white arrows in Fig. 4C and D indicate particles that are surrounded by LAMP-1-positive membranes and are therefore contained within the late phagosome/phagolysosome. Particles that were clearly not internalised by cells displayed no such ring of green fluorescence, thus demonstrating that the green fluorescence results from the LAMP-1-positive membrane rather than an artefact of overlapping fluorescent emission or direct binding of the LAMP-1 antibody to the particle.

Also present are particles that had been internalised by cells, but that did not display a ring of fluorescence. It is therefore likely that these particles were inside the phagosome, and that endosome/lysosome-phagosome fusion had not yet taken place. No difference was seen between the different shapes in terms of localisation. As in the case of the actin staining, the majority of particles were situated in the perinuclear region of the cell. Images of at least 40 cells from each sample were examined and the percentage of particles surrounded by LAMP-1-positive membranes calculated. The percentage of spheres and pyramids was similar (60.9 and 53.3%, respectively); however, the percentage of cuboids surrounded by LAMP-1-positive membrane was lower (31.0%).

### Quantitative determination of microchip uptake using imaging flow cytometry (IFC) and trypan blue quenching (TBQ) methods

#### Determination of microchip’s uptake in the absence and presence of LPS

RAW 264.7 macrophages were incubated with rhodamine B-labelled particle suspensions at particle:cell ratios of 5:1 and 10:1, with or without prior stimulation with LPS as macrophages are highly dynamic cells that exhibit phenotypic changes in response to environmental cues. The results are shown in Fig. S6. Determination of particle uptake was calculated in two ways. Firstly, the percentage of cells, which had internalised particles, was measured by counting the number of cells, which had a fluorescent intensity above the intensity of untreated cells and dividing by the total number of cells (Fig. S6A). Secondly, by fluorescent spot counting of cells with internal particles, the mean number of particles internalised per cell was calculated (Fig. S6B). The data in Fig. S6 shows the percentage of cells with internal particles indicates that there was no statistical difference between the two methods (IFC and TBQ) in any case. Owing to the way in which the IFC method discriminates internal from external particles (Figs. S7, S8, S9, S10, and S11 in supporting information), it might be expected that an over estimate of the percentage of cells with internal particles is observed. This is because although surface-bound particles can be discriminated in the *X*- and *Y*-planes, the depth of field in the *Z*-plane is not sufficient to distinguish whether particles are internal or surface bound. The TBQ method (Figures S12 and S13 in supporting information) does not suffer this drawback and instead relies on adequate fluorescence quenching to gate surface-bound particles. A study by Fei et al. [25] reported that it is not possible to suitably distinguish between internal- and surface-bound particles without the addition of a secondary fluorescent label. In contrast, a study by Vranic et al. [24] showed similar results between trypan blue quenching of fluorescein isothiocyanate (FITC)-labelled silica nanoparticles and imaging flow cytometry image analysis. Owing to the conflicting reports, it was necessary to assess the validity of this method for the particles used in this work by performing a comparison with a well-established trypan blue quenching method. The results of the current work agree with the study by Vranic et al. that showed equivalent uptake using TBQ and IFC [25]. A possible explanation for the lack of difference between the two methods is that the majority of particles are internalised rather than surface bound, and so the overestimation of surface-bound particles by the IFC method is minimised. Particle uptake at 4 °C was assessed using the IFC method (Fig. S14 in supporting information) and showed a reduction in particle uptake in comparison to 37 °C. Phagocytosis, an energy-dependent process, is reduced at this temperature, confirming that the IFC method was able to measure particle internalisation. The mean number of particles per cell was calculated and the data presented in Fig. S6B. In the majority of cases, IFC calculated a higher mean number of particles per cell than the TBQ method. This may result from the fact that surface-bound particles in cells that have both internal and external particles are included in the image analysis spot count but not in the trypan blue spot count (due to quenching).

RAW 264.7 cells were also stimulated with lipopolysaccharide (LPS) for 24 h prior to dosing in order to encourage differentiation to the M1 phenotype because macrophages are highly dynamic cells that exhibit phenotypic changes in response to environmental cues [42]. This was assessed visually by light microscopy as can be seen in Fig. S15 (supporting information). Cells that had undergone stimulation with LPS exhibited morphological changes as can been seen in Fig. S15B. In general, cells appeared larger and displayed vacuole-like and filopodia structures, indicative of M1-activated macrophages [43].

Particle uptake by cells was analysed using both IFC and TBQ, and the results are shown in Fig. S16 (supporting information). Figure S16A shows the effect of LPS stimulation on the percentage of cells that had internalised particles. LPS stimulation did not alter significantly the percentage of cells taking up particles. The only significant difference was observed in the case of spheres that were administered at a dose of 10 particles per cell and analysed using the TBQ method where LPS stimulation resulted in a significantly lower percentage of cells with internal particles. Similarly, there was very little difference in the average number of particles taken up per cell in LPS stimulated and unstimulated cells (Fig. S16B). The majority of cells internalised one or two particles with a much smaller proportion of cells internalising three or more particles. A study by Rieger et al. using IFC demonstrated that upon stimulation with LPS, RAW 264.7 macrophages association with microparticles increased overall; however, the cause for the increase in association was not an increase in internalisation but an increase in surface binding of zymosan particles [44].

**Fig. 5:**
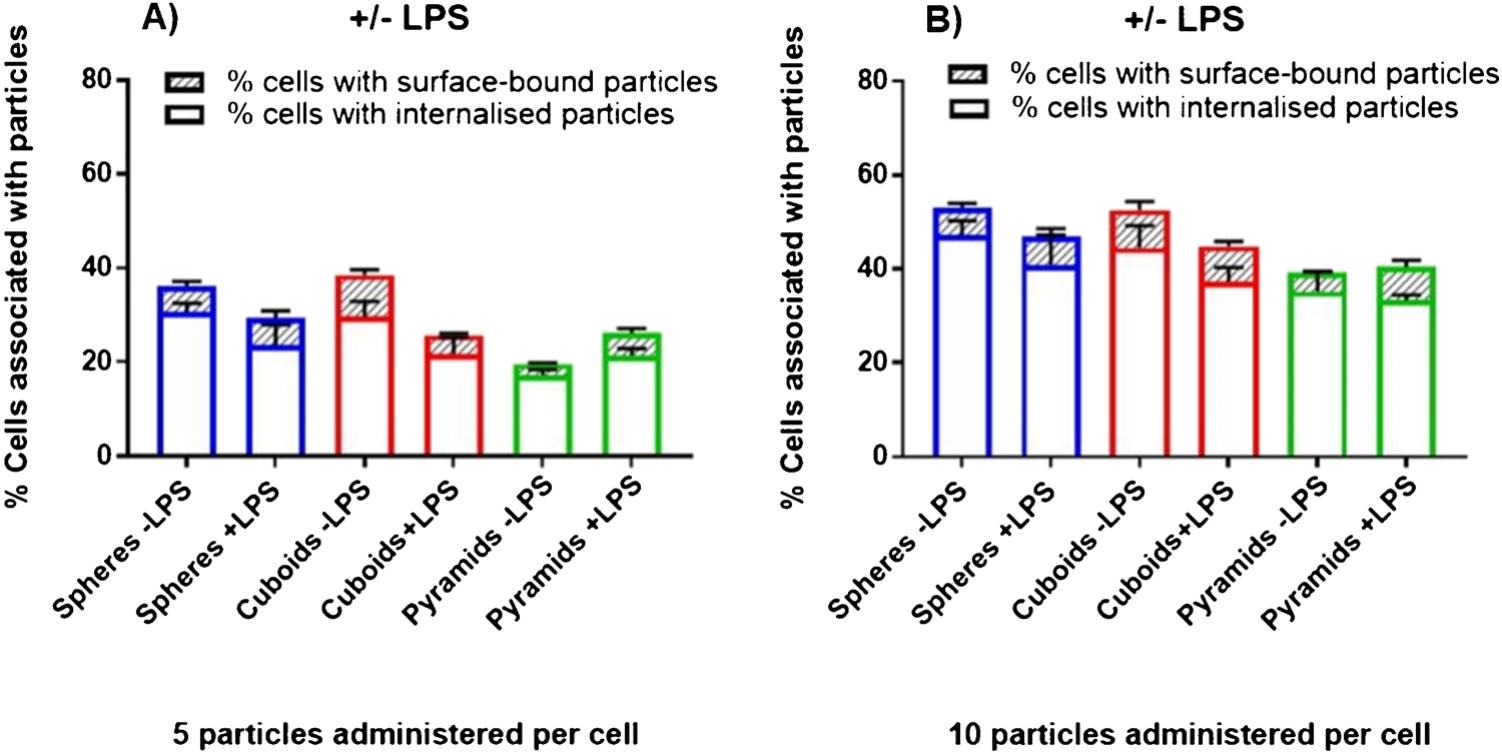
The effect of LPS stimulation on internalisation and surface binding for **A** 5 particles and **B** 10 particles administered per cells using the IFC method. *, **, ***, and **** indicate statistical significance (*p* < 0.05, 0.01, 0.001, 0.0001, respectively) as calculated by multiple unpaired *T*-tests. Values are representative of three biological repeats ± SEM (*N* = 3)

If the same was true in the case of the silicon oxide particles used in this study, the increased binding of particles to the cell surface would not be detected by the TBQ method as these additional particles would be quenched. The IFC method can quantify this effect by analysing the percentage of cells with surface-bound particles as shown in Fig. 5. At a dose five particles per cell, the results indicate that approximately 30% of the cells have internalised spheres and cuboids. However, the percentage of cells with surface-bound spheres and cuboids is about 5% and 10%, respectively. In the case of pyramids, approximately 20% of cells take up the microchips, and about 5% of cells have surface-bound microchips. When the ratio of particles per cell was doubled, the percentage of cells with internalised spheres and cuboids increased to around 50% and that of pyramids to about 35%. On the other hand, the percentage of cells with surface-bound spheres and cuboids was ca. 5% and 10%, respectively, and that of pyramids to about 5%. Higher surface binding was not observed upon stimulation with LPS; instead, no statistically significant difference in overall particle association was observed in LPS-stimulated cells.

#### Effect of microchip shape on uptake by RAW 264.7 macrophages

**Fig. 6:**
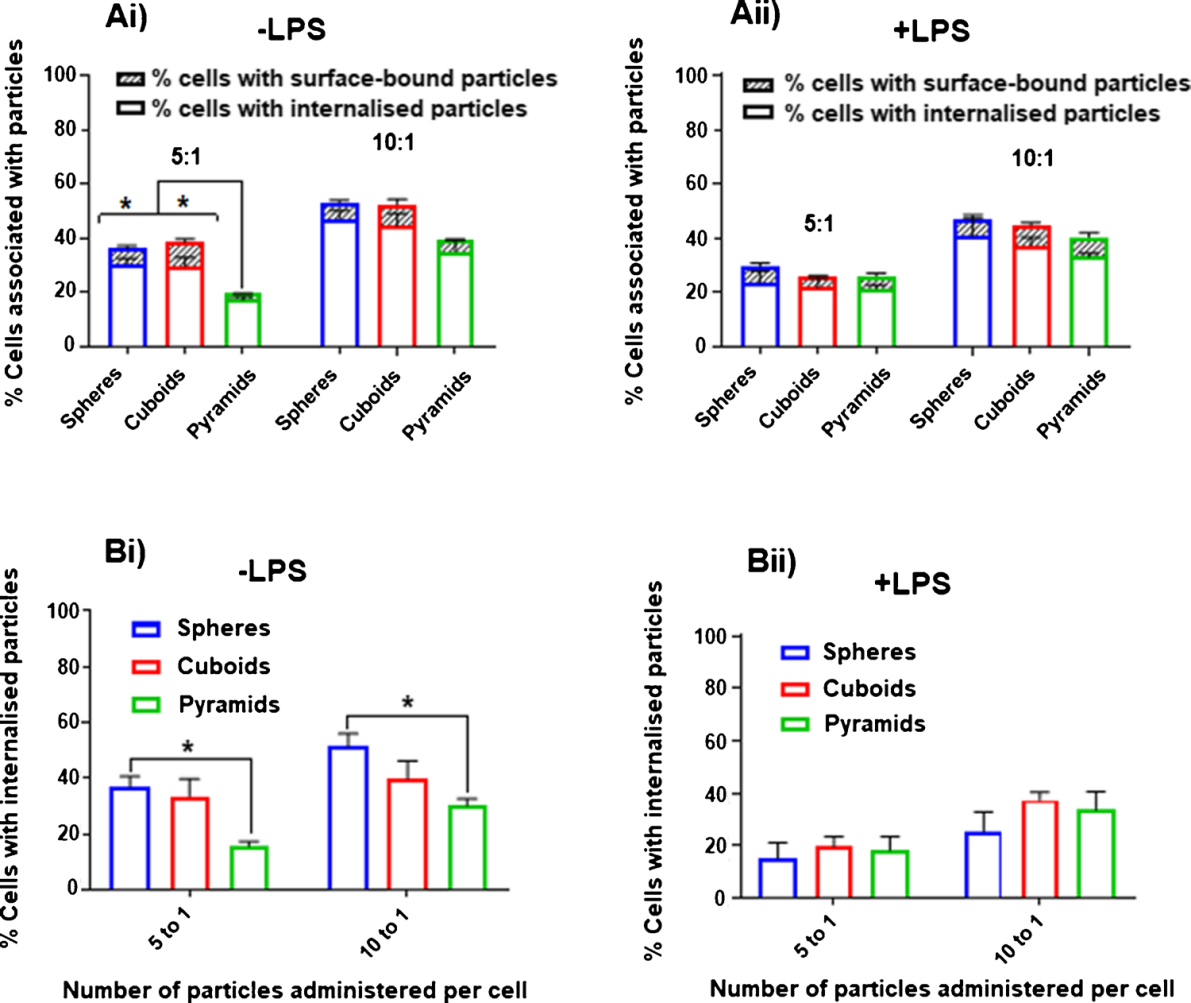
The effect of particle shape on cellular uptake. The effect of particle shape on % of cells with internal particles **Ai** and **Aii** analysed using IFC and **Bi** and **Bii** analysed using TBQ method. *, **, ***, and **** indicate statistical significance (*p* < 0.05, 0.01, 0.001, 0.0001, respectively) as calculated by two-way ANOVA with multiple comparisons. Values are representative of three biological repeats ± SEM (*n* = 3)

Macrophages have previously been shown to display differential uptake of polymer nanoparticles depending on their morphology [45–47]. As such, it was expected that differences in the internalisation of spheres, cuboids, and pyramids would be observed. The results from analysing differences in internalisation between the different particle shapes using both IFC and TBQ methods are shown in Fig. 6. Using both analysis methods, an increase in the percentage of cells with internalised particles was observed as the particle dosage per cell doubled from 5 to 10 particles per cell (Fig. 6). This effect was seen in both LPS-stimulated and non-stimulated cells. However, the mean number of particles internalised per cell with the average of 1–1.5 particle/cell did not change with increasing the dose of particles per cell (Fig. S17). Using the IFC, the data is presented as the percentage of cells associated with particles and further distinguishes the percentage of cells with internal and surface-bound particles (Fig. 6Ai, ii). In this case, no significant differences between spheres and cuboids in terms of the percentage of cells associated with particles, the percentage of cells with internalised particles, or the percentage cells with surface-bound particles. This was the case for both LPS-stimulated and non-stimulated cells. In addition, analysis by TBQ shows no significant differences in internalisation of particles between spheres and cuboids (Fig. 6Bi, ii). However, significantly fewer cells internalised pyramids compared to spheres indicating that the ability of macrophages to internalise pyramids is lower. This may result from the anisotropic nature of these particles, which can present to cells in multiple orientations as opposed to spherical particles, which can only make contact with the cell in one orientation. Actin polymerisation must then occur in order to wrap the plasma membrane around the particle, and differences in the capacity of membrane wrapping, depending on particle orientation, could therefore result in reduced uptake.

Curiously, this effect was seen only in cells that had not been stimulated with LPS. There were no differences between any of the shapes in LPS-stimulated cells. Filopodia and membrane ruffles on the surface of macrophages are dynamic structures which have been shown to contribute to macropinocytosis and phagocytosis. Upon stimulation with LPS, actin polymerisation is increased resulting in more filopodia and membrane ruffles [48]. This change in the macrophage skeletal structure could therefore explain the reduced the effect of particle shape seen in LPS-stimulated cells. No differences were seen in the number of particles internalised per cell for any particle shape (Fig. S17). The majority of cells that internalised particles only internalised one particle regardless of particle shape, and this was consistent for both particles’ doses. In a similar study by Sharma et al. which looked at the internalisation of polystyrene spheres, oblate ellipsoids, and prolate ellipsoids by RAW 264.7 macrophages, there was no difference in internalisation seen between the different shapes with volumes above 24 μm^3^ (particles stretched from a 3.6-μm Φ sphere) [45]. There were, however, significant differences seen between the shapes that had volumes up to 0.69 μm^3^. The volumes of the particles used in this work were 14.1 μm^3^ (spheres), 11.6 μm^3^ (cuboids), and 17.2 μm^3^ (pyramids) and so are in between the volumes of particle groups used by Sharma et al. On the other hand, shape effects on internalisation of larger microparticles by macrophages have been reported by Doshi et al. who found that hyaluronic acid-coated disk-like microparticles (diameter = 6 μm) were able to avoid uptake by J774 macrophages compared with spherical particles (6 μm) of the same surface composition which exhibited high uptake [46]. Additionally, Champion and Mitragotri have shown that worm-like particles (aspect ratio > 20, length ~ 10 μm) are internalised significantly less that spheres of equivalent volume [47].

## Conclusions

Silicon oxide microchips of different morphologies — either commercially available spheres or microfabricated rectangular cuboids and apex-truncated square pyramids — have been fluorescently labelled in order to study their interaction with living cells. Both *surface*- and *in suspension*-controlled covalent functionalization have resulted in a uniform, reduced — 2% of surface coverage per microchip — and stable labelling of the microchips. Imaging flow cytometry (IFC) has been used as a method for quantifying microparticle uptake, compared with the trypan blue quenching method and proved to give similar results demonstrating its usefulness as a technique which does not rely on specific quenching of fluorophores. The effect of particle shape on cellular uptake was evaluated. RAW 264.7 cells could phagocytose all particle shapes, which were shown to follow the established phagosomal maturation pathway. The uptake of particles increased with increasing dose and was not significantly altered upon stimulation of macrophages with LPS. There was a negligible difference in the uptake of particles between spheres and cuboids, regarding both the percentage of cells containing internalised particles and the number of particles internalised per cell. The findings reveal that at a rate microchip:cell of 5:1, ca. 30% of the cells internalise spheres and cuboids, with the average of 1–1.5 particle/cell, whereas the proportion of cells with surface-bound spheres and cuboids is approximately 5% and 10%, respectively. However, pyramids demonstrated a decreased uptake about 20% of cells engulf the microchips and the same average of 1–1.5 particle/cell, while around 5% have surface-bound microchips. When the particle dose was doubled to 10:1, an increase to ca. 50% of the percentage of cells with internalised spheres and cuboids was observed, whereas for pyramids it was ca. 35%. Moreover, the percentage of cells with surface-bound spheres and cuboids was approximately 5% and 10%, respectively, and for pyramids it was about 5%. However, the number of microchips internalised per cell did not change after increasing the dosage of particles, and it was ca. 1.5 microchip/cell, a remarkable value considering the overall size of the microchips.

Overall, the microfabricated cuboids show a comparable rate of internalisation to the widely studied and commonly used spheres. This suggests that cuboids could be viewed as superior choices for micro-carrier applications because they enable controlled multi-functionalization in terms of space, for example through micro-contact printing techniques. In addition, cuboid microchips offer several advantages over spherical particles, making them superior in biomedical applications. The microfabrication of cuboid microchips allows for precise size and shape control, avoiding the issue of polydispersity commonly seen with spherical particles. This precise control also enhances their versatility and morphology, enabling tailored designs for the biological applications. The high surface area of cuboid microchips supports high loading capacities and facilitates chemical functionalization, improving their functionality. Furthermore, cuboid microchips are biocompatible, easier to observe under microscopic analysis, and exhibit prolonged retention within cells, often accumulating in targeted tissues. These characteristics collectively make cuboid microchips a more effective and reliable option for biomedical applications.

## Supplementary Information

Below is the link to the electronic supplementary material.

Supplementary file1 (DOCX 5156 KB)

## Data Availability

The datasets generated during and/or analysed during the current study are available from the corresponding author on reasonable request.
