# Supplementary material for: Exploring the influence of silicon oxide microchips shape on cellular uptake using imaging flow cytometry
Source: Mikrochim Acta. 2024 Aug 21;191(9):554. doi: 10.1007/s00604-024-06631-7 (PMC11339096; doi:10.1007/s00604-024-06631-7)
Supplement: Supplementary file 1 — Supplementary file1 (DOCX 5156 KB) [file 604_2024_6631_MOESM1_ESM.docx]

**SUPPORTING INFORMATION**

**Exploring the influence of silicon oxide microchips shape on cellular uptake using imaging flow cytometry**

Gordon Bruce,^1^ Saman Bagherpour,^2,3^ Marta Duch,^4^ José Antonio Plaza,^4^ Snow Stolnik,^5^ Lluïsa Pérez-García^1,2,3^*

*^1^ Division of Advanced Materials and Healthcare Technologies, School of Pharmacy, University of Nottingham, Nottingham NG7 2RD, UK*

*^2^ Departament de Farmacologia, Toxicologia i Química Terapèutica, Facultat de Farmàcia i Ciències de l’Alimentació, Universitat de Barcelona, Av. Joan XXIII 27-31, 08028 Barcelona, Spain*

*^3^ Institut de Nanociència i Nanotecnologia (IN2UB), Universitat de Barcelona, 08028 Barcelona, Spain*

*^4^ Instituto de Microelectrónica de Barcelona IMB-CNM (CSIC), Campus UAB, Cerdanyola del Vallès, Barcelona 08193, Spain*

*^5^ Division of Regenerative Medicine and Cellular Therapies, School of Pharmacy, University of Nottingham, Nottingham NG7 2RD, UK*

**Experimental**

**1 Materials**

Fluoromount™ F4680, Rhodamine B isothiocyanate (RBITC, 283924), 3-aminopropyltrimethoxysilane (APTMS, 281778), 4-(2-hydroxyethyl)-1-piperazineethanesulfonic acid (HEPES H0887), Hank’s Balanced Salt Solution (HBSS, H8264), silicon oxide spheres (diameter 3 μm), Dulbecco’s Modified Eagle Medium (DMEM, D546), Foetal bovine serum (FBS, F7524), L-glutamine (G7513), Triton X-100 (TX-100), 4’,6-diamidine-2’-phenylindole dihydrochloride (DAPI), fluoroshield™ (F6182), and Penicillin/Streptomycin were purchased from Sigma-Aldrich. H_2_SO_4_ (98%), NH_4_OH (20%), Na_2_HPO_4_/NaH_2_PO_4_ buffer 8, ethanol (EtOH), and acetone were purchased from Fisher Scientific. 11-Aminoundecyltriethoxysilane (AUTES, S25045) was purchased from Fluorochem (UK). H_2_O_2_ (35%), formaldehyde 4%, and dimethylsulfoxide (DMSO) were purchased from VWR. BDP-630 NHS ester was also purchased from Lumiprobe. Phosphate-buffered saline (PBS) tablets were obtained from Oxoid Ltd (UK). Phallodin Ifluor 488 (ab176753) was also purchased from Stratech Scientific. Tissue culture treated 75 cm^2^ (T-75) cell culture flasks was purchased from Corning life sciences (Holland). Lipopolysaccharide (LPS), CD107a (LAMP-1) monoclonal antibody, Alexafluor 488, and Accutase were purchased from Fisher Scientific. Trypan blue (TB) was purchased from Sigma-Aldrich. Milli-Q water produced by a MilliQ plus system from Millipore.

**2 General methods**

Water contact angle measurements were acquired using a KSV CAM 200 optical contact meter. Surfaces were placed with the silicon oxide particle face upwards and the water contact angle measured. Measurements were taken in triplicate (3 wafer pieces per sample). Brightfield and fluorescence images were acquired using a Nikon Eclipse TiU fluorescence microscope. The number of particles per mL was calculated by counting particles on a haemocytometer (Scientific Laboratory Supplies (UK)). Confocal microscopy was performed using a Zeiss Elyra PS 1 LSM780 inverted confocal microscope with a Zeiss 63x water immersion objective. Excitation/emission wavelengths for each fluorophore are as follows: DAPI (λ_ex_: 360 nm, λ_em_: 450 nm) Ifluor 488 (λ_ex_: 480 nm, λ_em_: 520 nm) Alexa Fluor 488 (λ_ex_: 480 nm, λ_em_:520 nm) BDP 630 (λ_ex_: 630 nm, λ_em_: 650 nm). Images were processed using ImageJ®[37]**.** All flow cytometry was performed using an Amnis imagestream^X^ MKII imaging flow cytometer in standard configuration with 40X magnification. Illumination settings: Brightfield LED 32.01 mW, 561 nm laser 200 mW, 785 nm laser 2 mW. Data was acquired using INSPIRE software with a minimum of 500 cells per sample (typically > 1000 per sample). Data was analysed using IDEAS software.

**3 Acquisition of brightfield and fluorescent images**

Images of wafer pieces were acquired by placing the wafer piece on a glass slide with the silicon oxide side face-down. Images of particles in suspension were acquired by pipetting 15 μL particle suspension onto a glass slide and then covering with a coverslip. For fluorescence imaging of RBITC labelled samples λ_ex_ = 550 nm and λ_em_ >590 nm. Images were processed using ImageJ® to produce fluorescent surface plots. For fluorescence imaging of BDP 630 labelled particles λ_ex_ =630 nm and λ_em_ >670 nm.

**4 Routine cell culture methods**

**4.1 Culture of RAW 264.7 cells**

RAW 264.7 macrophages were cultured in T-75 cell culture flasks. 15 mL high glucose DMEM supplemented with 10% FBS, 1% L-glutamine, and 1% penicillin/streptomycin was used as the culture media. Cells were incubated at 37 °C with 5% CO_2_ in 95% humidity. Cell culture media was removed by aspiration and replaced with 15 mL pre-warmed fresh media every 48 hours.

Cells were harvested by gentle scraping once they reached 60-80% confluency. Successful detachment of cells by scraping was confirmed by examination under a microscope. Detached cells in media were transferred by pipette into a 15 mL falcon tube. Falcon tubes containing cells were centrifuged at 1200 RPM (300G) for 5 minutes to cause the cells to form a pellet. The supernatant was removed by aspiration and replaced with 6 mL fresh (pre-warmed at 37 °C) media. The cells were suspended in the fresh media by pipetting up and down to disperse the pellet. At this point, cells would either be counted for seeding in well plates for experiments, or if undergoing routine passaging, would be split into new T-75 culture flasks containing 15mL fresh pre-warmed media. Typical passaging ratios from 6 mL cell suspension were 1:3 and 1:6. Cells were passaged between P13 and P30 after which cells were disposed of and fresh P13 cells were revived from storage.

**4.2 Cell counting, freezing, and revival**

A haemocytometer was used to count the number of cells for each experiment. 10 μL detached cell suspension in fresh pre-warmed media was transferred to a 200 μL microcentrifuge tube. To this, trypan blue solution (0.4%, 10 μL) was added and mixed by pipetting the solution up and down. 10 μL of this mixture was transferred onto the haemocytometer and cells counted under the microscope. Cells were counted from the four corners of the haemocytometer and the sum divided by four to give an average. This result was then multiplied by 2 to account for the dilution by adding the trypan blue solution and then multiplied by 10,000 to give the number of cells per 1 mL.

After detachment of cells and centrifugation cells were suspended in 1mL cell culture media containing 10% DMSO. This cell suspension was then transferred by pipette to a 1 mL cryovial. Cryovials were labelled with the date, cell type, and passage number and then placed in a Mr Frosty™ (Thermoscientific) freezing container containing isopropanol. The Mr Frosty™ container was placed in a -80 °C freezer overnight after which time cryovials were transferred into liquid nitrogen containers for long-term storage. Each cryovial contained a whole flask of cells.

After removal of a cryovial containing cells from liquid nitrogen storage, the cryovial was placed in a water bath at 37 °C until partially defrosted. As soon as the cell suspension had partially defrosted, it was transferred to a 15 mL falcon tube and 10 mL pre-warmed media added. The suspension was centrifuged at 1200 RPM (300 G) for 5 minutes and the supernatant removed. The pellet was suspended in 15 mL fresh pre-warmed media and this cell suspension transferred to a fresh T-75 cell culture flask and stored in an incubator at 37 °C, 95% humidity, 5% CO_2_.

**5 Immunofluorescence staining and preparation of confocal slides**

Actin staining was performed by addition of 1 mL per well pre-warmed Ifluor 488 phalloidin solution in PBS (1 μL ifluor 488 stock solution in 1 mL PBS) for 30 minutes at room temperature while protected from light. Staining solution was then removed and the cells washed with PBS (3×1 mL). Alternatively, LAMP-1 staining was achieved by the addition of 1 mL per well of Alexa Fluor 488 labelled CD107a antibody (5 μg per mL in PBS) for 45 minutes while protected from light. Cells were washed with PBS (3×1 mL). Nuclear staining was achieved by addition of 1 mL per well pre-warmed 300 nM DAPI solution in PBS for 5 minutes at room temperature. DAPI solution was then removed by aspiration and the cells washed with PBS (3×1 mL).

Coverslips were removed from the well plate and rinsed with deionised water to prevent salt crystal formation and mounted onto glass slides with a drop of fluoroshield™ mounting medium. Slides were sealed using nail varnish and stored protected from light at 4 °C. Confocal images were obtained within one week of slide preparation.

**6 Using spot count analysis to calculate the number of microchips per cell**

Populations of cells with internal particles (as determined using both the imaging flow cytometry and the trypan blue quenching methods) were subsequently analysed using the IDEAS analysis software spot count feature to determine the number of particles per cell.

**Table S1**. Dimensions of fabricated cuboids, pyramids, and spheres.

|  | **Height**  **(μm)** | **Base Width**  **(μm)** | **Top Width**  **(μm)** | | | **Diameter**  **(μm)** | **Surface Area (μm^2^)** | | | **Volume (μm^3^)** |  |
| --- | --- | --- | --- | --- | --- | --- | --- | --- | --- | --- | --- |
| **Cuboids** | 1.1 | 3.4 | | 3.1 | - | | | 35.1 | 11.6 | | |
| **Pyramids** | 3.2 | 2.8 | | 1.8 | - | | | 40.5 | 17.2 | | |
| **Spheres** | - | - | | - | 3 | | | 28.3 | 14.1 | | |


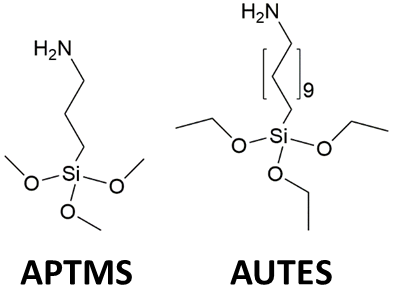


**Fig. S1**. a) Chemical structures of silanes: 3-aminopropyltrimethoxysilane (APTMS) and 11-aminoundecyltriethoxysilane (AUTES)

**Table S2**. Water contact angles θ (°) on the silicon oxide surfaces after treatment with APTMS, and AUTES

| **Surface treatment** | **Water contact angle** ± SD^1^ |
| --- | --- |
| Activation | < 10° |
| APTMS | 38° ± 11 |
| AUTES | 62° ± 6 |

^1^SD: Standard deviation


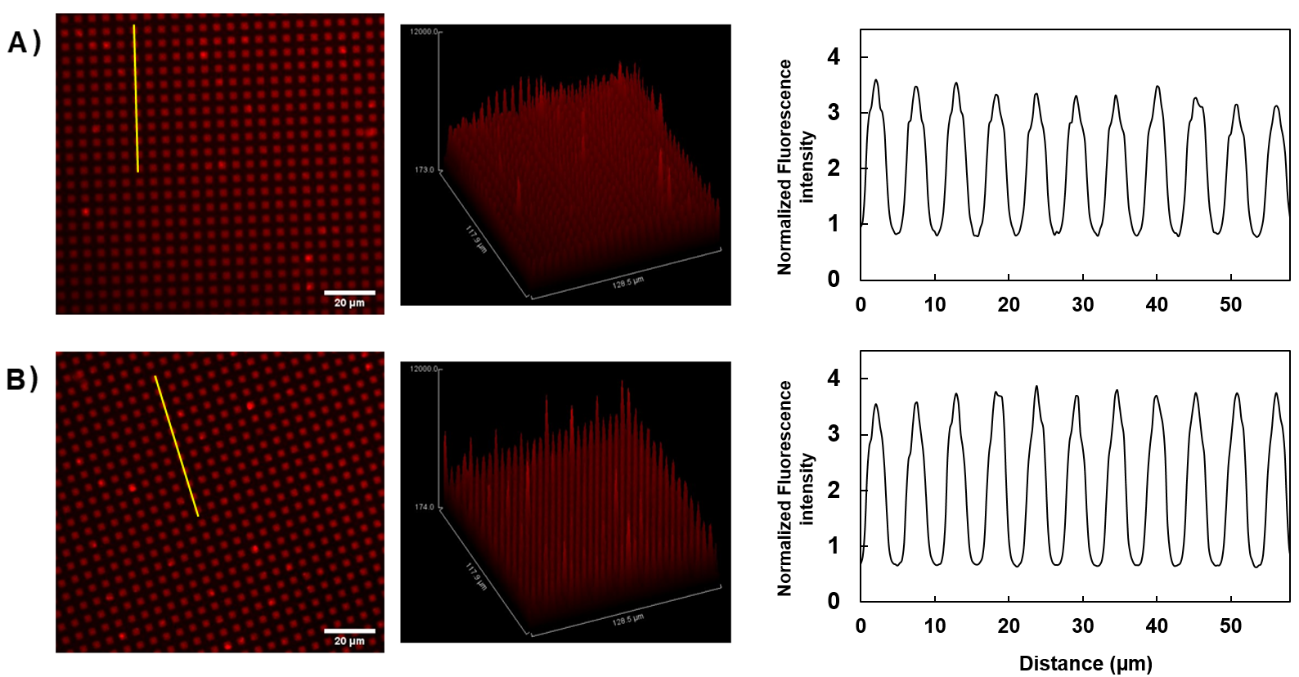


**Fig. S2**. Images of particles in wafers before and after incubation in PBS 37 °C for 2 hours. 2D Fluorescence images(left), fluorescence surface plots (center), and fluorescence intensity profiles of particles (yellow line at left image) before (A) and after (B) incubation in PBS for 2 hours at 37 °C for particles labelled with AUTES and RBITC.


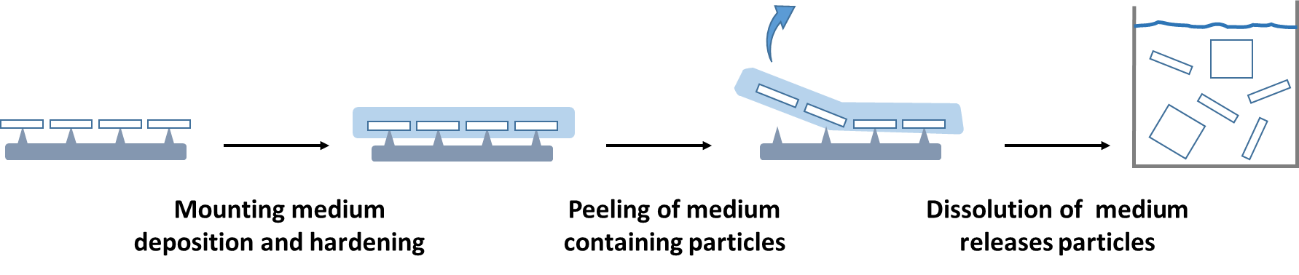


**Scheme S1**. Peeling of microchips from silicon wafer

*
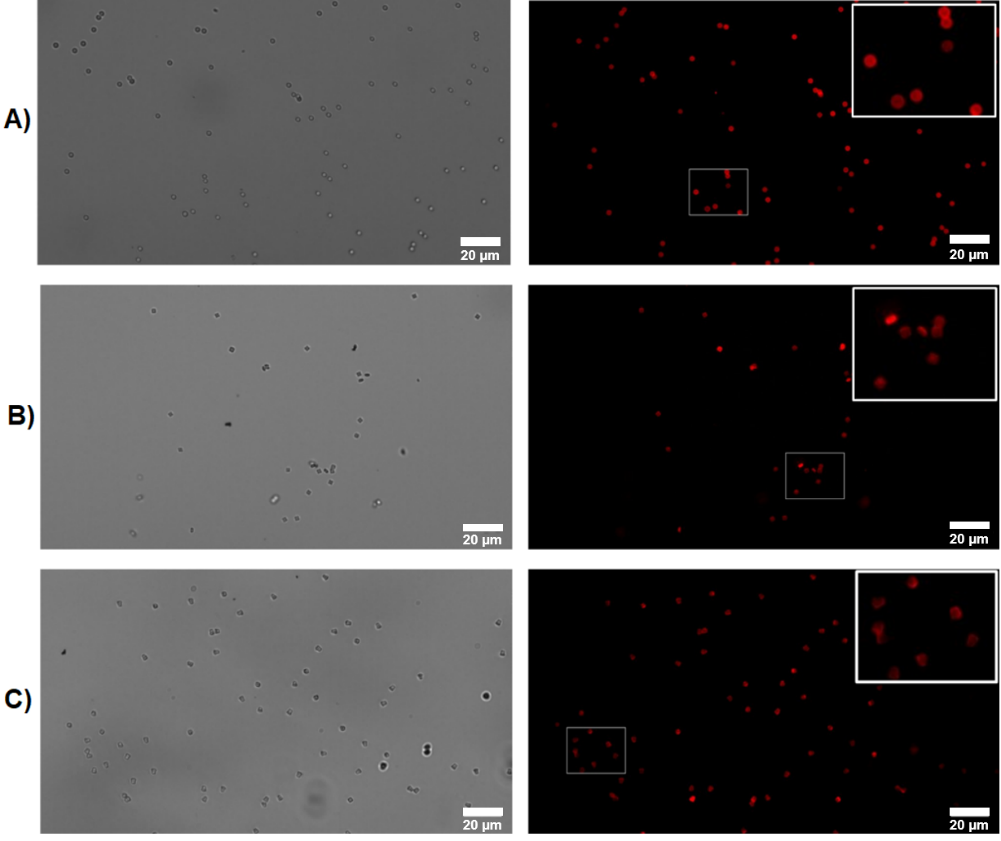
*

**Fig. S3**. Fluorescently labelled particles in suspension. Brightfield (left) and fluorescence images(right) of A) Spheres B) Cuboids C) Pyramids after immobilisation of AUTES and labelling with RBITC.

(1)

| **Particle type** | **Particle MFI** | **RBITC-labelled surface %** | **Quenched particle MFI** | **Quenching efficiency (%)** |
| --- | --- | --- | --- | --- |
| Spheres | 21477 | 1.84 | 1922 | 91.05 |
| Cuboids | 22276 | 1.92 | 987 | 95.57 |
| Pyramids | 18095 | 1.53 | 1062 | 94.13 |

(2)


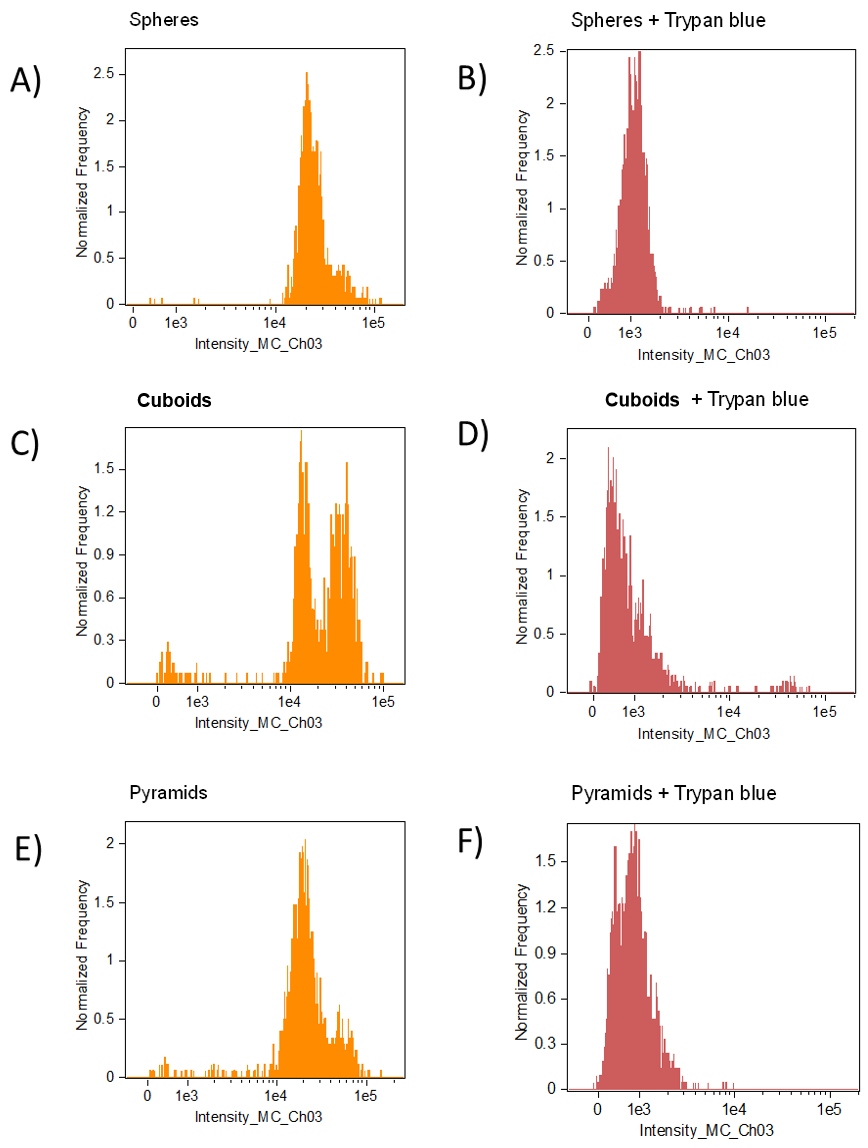


**Fig. S4**. (1) Particle MFI, estimation of surface labelling, and Quenching efficiency of trypan blue. (2) Fluorescence intensity histograms of RBITC labelled particles before and after trypan blue quenching*.* A) Spheres B) Spheres + trypan blue C) Cuboids D) Cuboids + trypan blue E) Pyramids F) Pyramids + trypan blue.


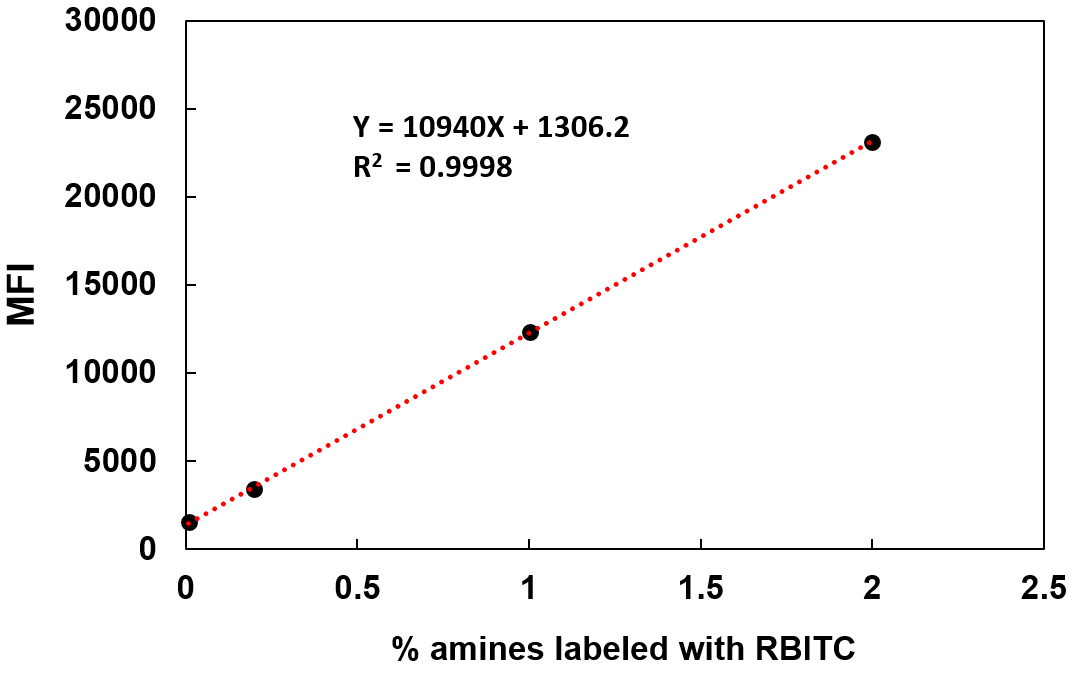


**Fig. S5**. Calibration of particle labelling. 3 μm silicon oxide spheres were modified using AUTES pre-labelled with different concentrations of RBITC so that the percentage of surface labelling was known. MFI was then measured using flow cytometry to compare with silicon oxide shapes labelled using AUTES then RBITC sequentially.


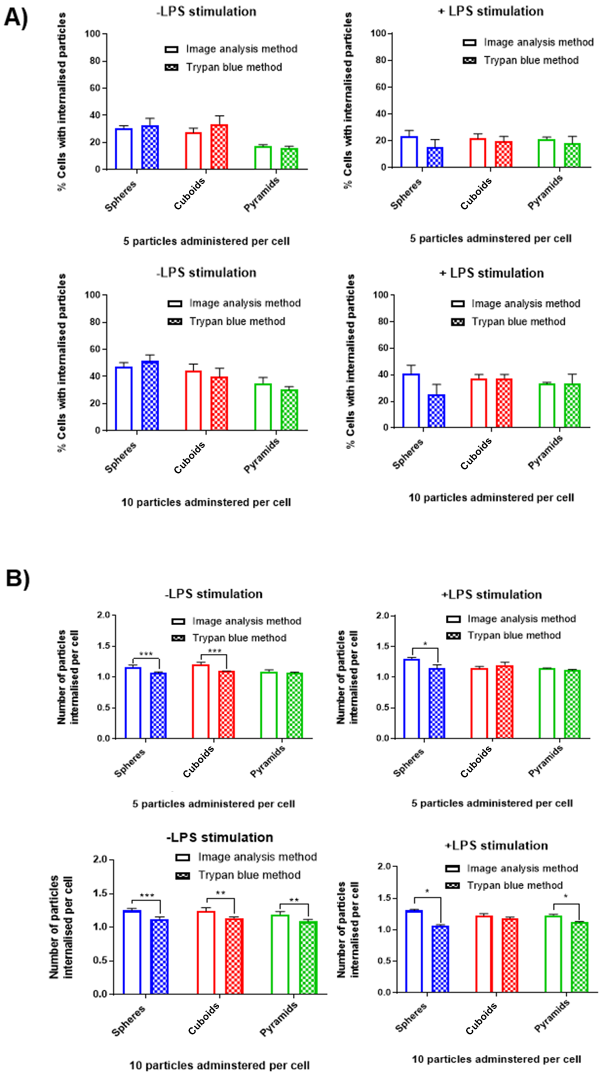


**Fig. S6**. Comparison of IFC and TBQ methods for quantifying particle internalisation. A) % Cells that had internalised particles. B) Number of particles internalised per cell. *, **, ***, **** indicate statistical significance (p < 0.05, 0.01, 0.001, 0.0001 respectively) as calculated by multiple unpaired T-tests. Values are representative of three biological repeats ± SEM (N=3).

**Quantification of microchip internalisation by imaging flow cytometry (IFC)**

Single cell populations were determined by plotting cell area against cell aspect ratio as shown in Figure S6. Each dot on the scatter plot is linked with an image and so gating can be checked visually and different populations distinguished to remove unwanted images from the analysis. The population of single cells is then analysed to select a population of cells that are in focus. This is done by creating a histogram using the gradient RMS feature as shown in Figure S8. This feature ranks images based on their focus, images that have higher scores are more in-focus. By visually checking cells in each bin, a minimum focus can be designated (GRMS > 50) which is then used for all further analyses.


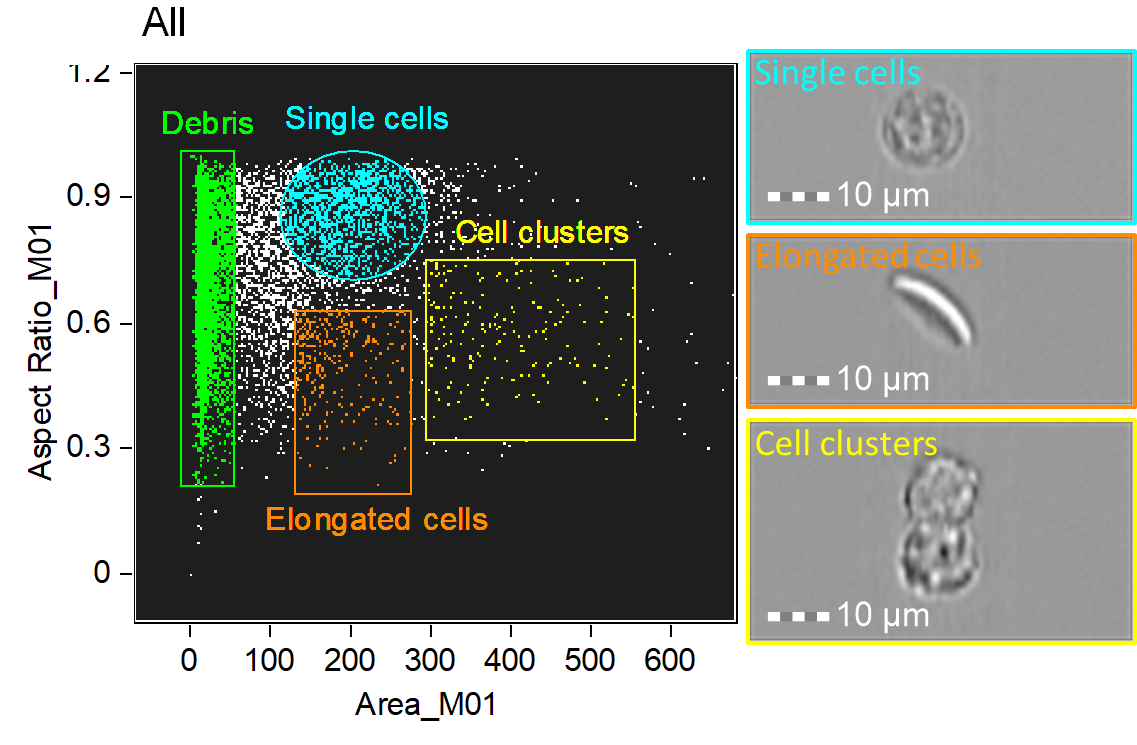


**Fig. S7**. Gating of single cell population

**
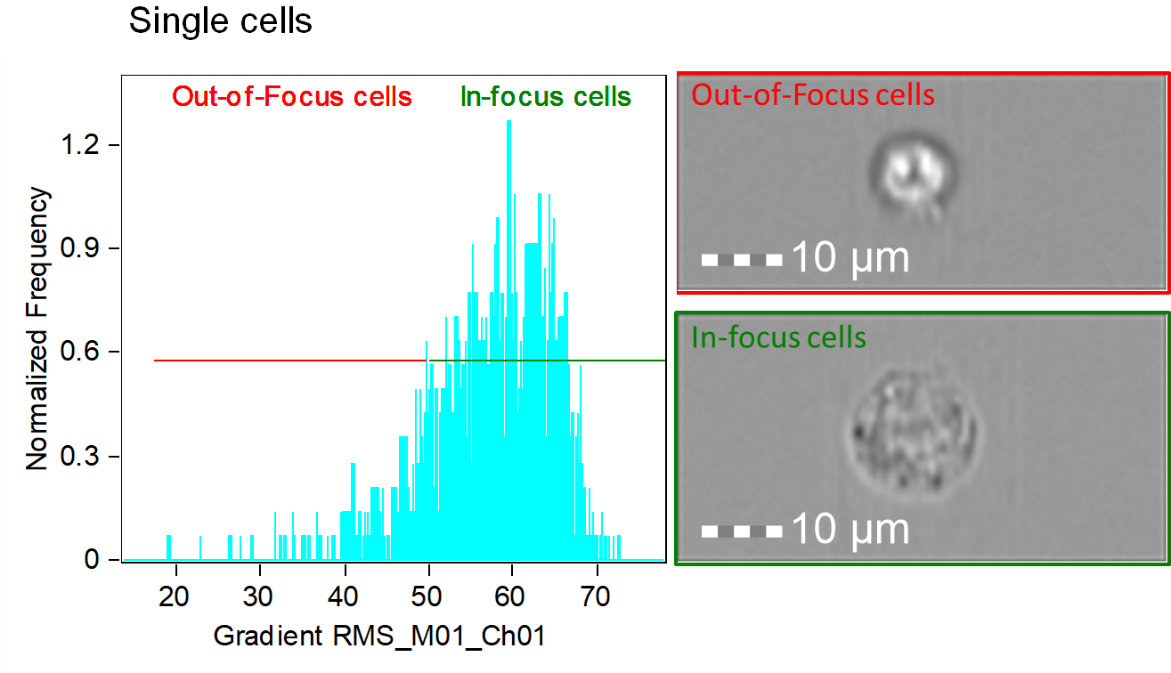
**

**Fig. S8**. Gating of in-focus cell population

In order to quantify particle uptake using IFC, it was first necessary to distinguish populations of cells which were associated with particles. This was achieved by plotting a histogram of fluorescence intensity as shown in Figure S9. To adequately distinguish populations, a control sample of cells that had not been exposed to particles was analysed and a region spanning the fluorescence range of these cells designated as non-associated cells (Figure S9A). A region with a fluorescence intensity above this gate was then designated as being associated with particles. By analysing a sample of cells which had been exposed to fluorescent particles, it was demonstrated that a distinct population appears within the associated region (Figure S9B). Further confirmation of the cells association with particles was performed by selecting images from each of the region’s examples of which are shown in Figure S9C. Particle fluorescence is present only in associated cells.


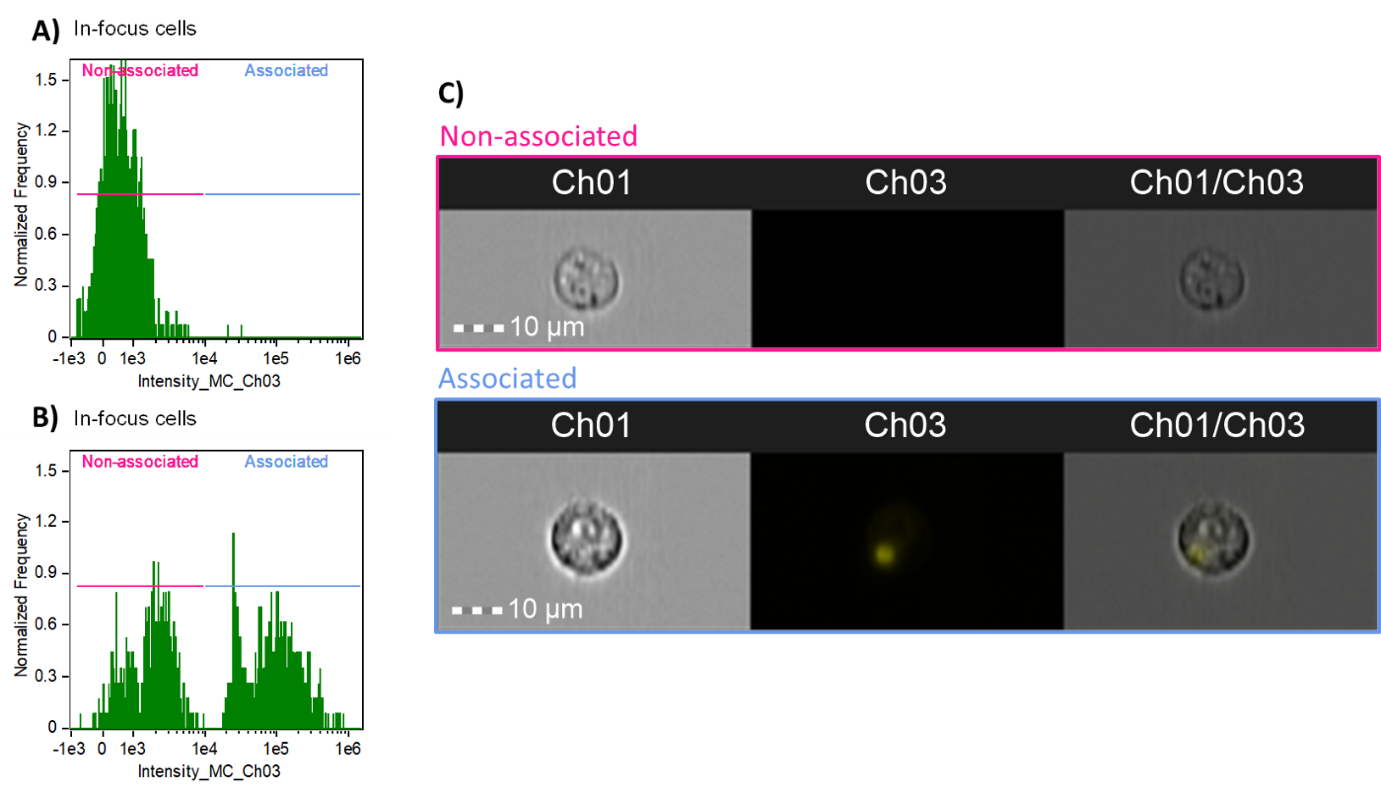


**Fig. S9**. Gating of cells associated with particles. A) Histogram of untreated cells, B) histogram of cells treated with RBITC-labelled silicon oxide spheres, C) Images of cells from non-associated and associated populations

To distinguish surface bound particles from internalised particles, a cell mask was created as shown in Figure S10A (blue region). This mask was eroded to exclude the cell membrane using the adaptive erode feature with an adaptive erode coefficient of 80 as shown in Figure S10B. This defines an area specific to each cell outside of which particles are considered surface bound by considering the ratio of fluorescence intensity inside the mask to the intensity of the entire cell. This analysis gives each image an internalisation score where a positive score indicates the particle is inside the cell and a negative score indicates the particle is surface bound. A histogram displaying the internalisation scores is produced and regions defined as “cells with external particles” and “cells with internal particles” (Figure S11).


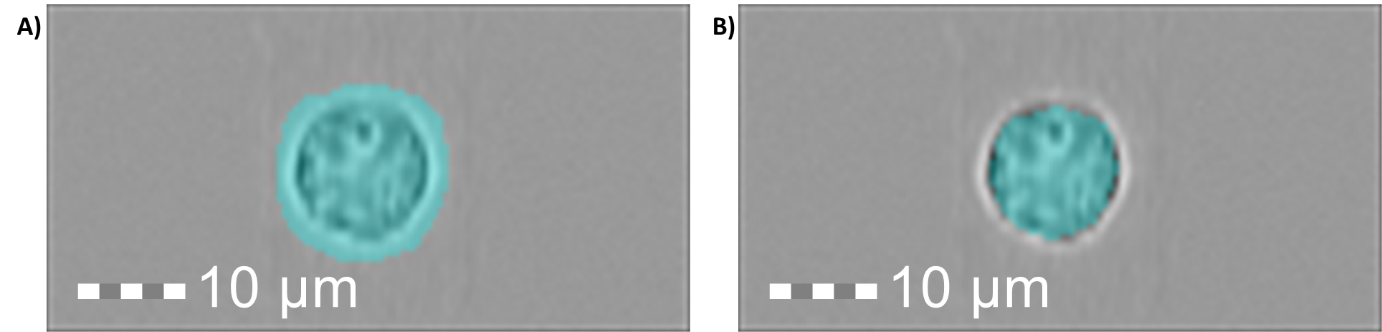


**Fig. S10**. Cell masks used to identify cells with internal and surface-bound particles. A) General cell mask B) Eroded cell mask

**
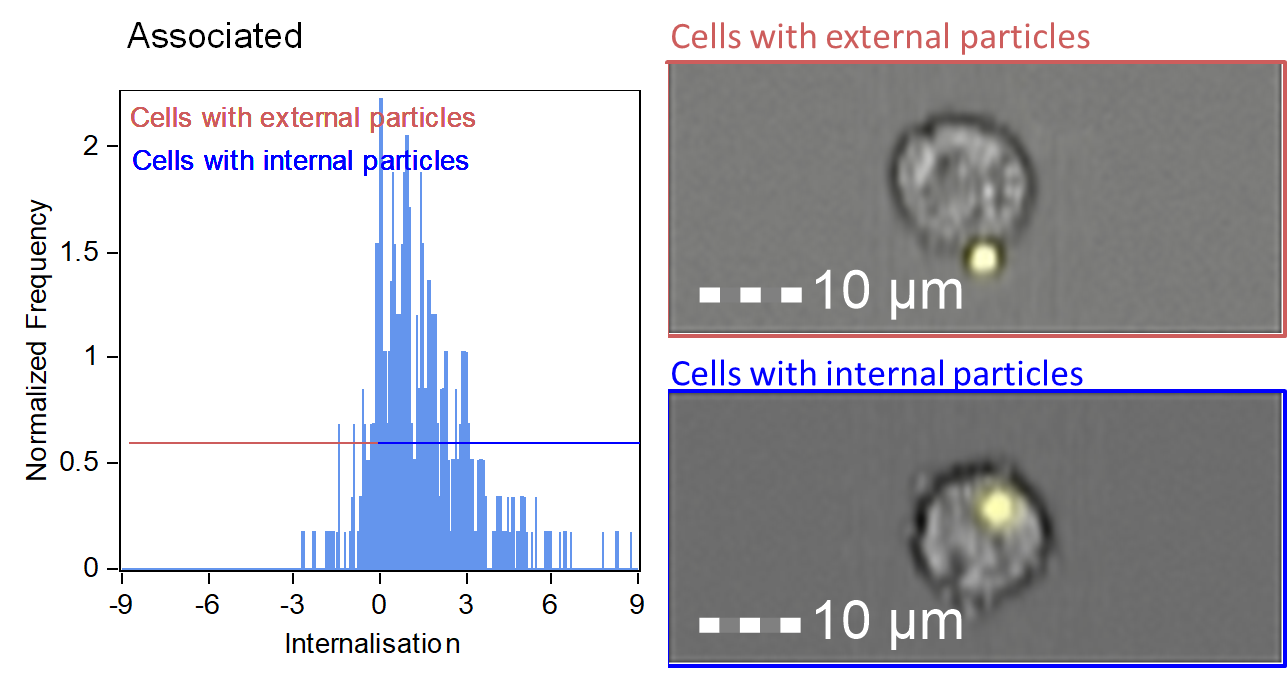
Fig. S11**. Histogram of internalisation score defining whether particles are internalised or surface-bound

**Quantification of microchip internalisation by trypan blue quenching (TBQ)**

The trypan blue quenching method distinguishes internalised particles from those, which are surface bound by quenching the fluorescence of the surface-bound particles. Trypan blue is a cell-impermeable dye that only enters the cell when the membrane is damaged and is therefore normally used to distinguish live and dead cells. Trypan blue has an absorption maximum at 580 nm which is the same wavelength of the emission of rhodamine B and so it quenches fluorescence when in close contact with the fluorophore by absorbing emitted light. As such, only those particles which it can come into close contact with i.e. surface bound particles are quenched.

After following the initial analysis step to isolate single cell populations as in figure S4, a plot of intensity in brightfield channel-Ch09 (X-axis) against intensity of fluorescence channel-Ch03 (Y-axis) is used to distinguish cell populations. The brightfield channel intensity relates to the degree of trypan blue staining, dead cells appear darker, and so this can be used to distinguish live from dead cells (Figure S12).


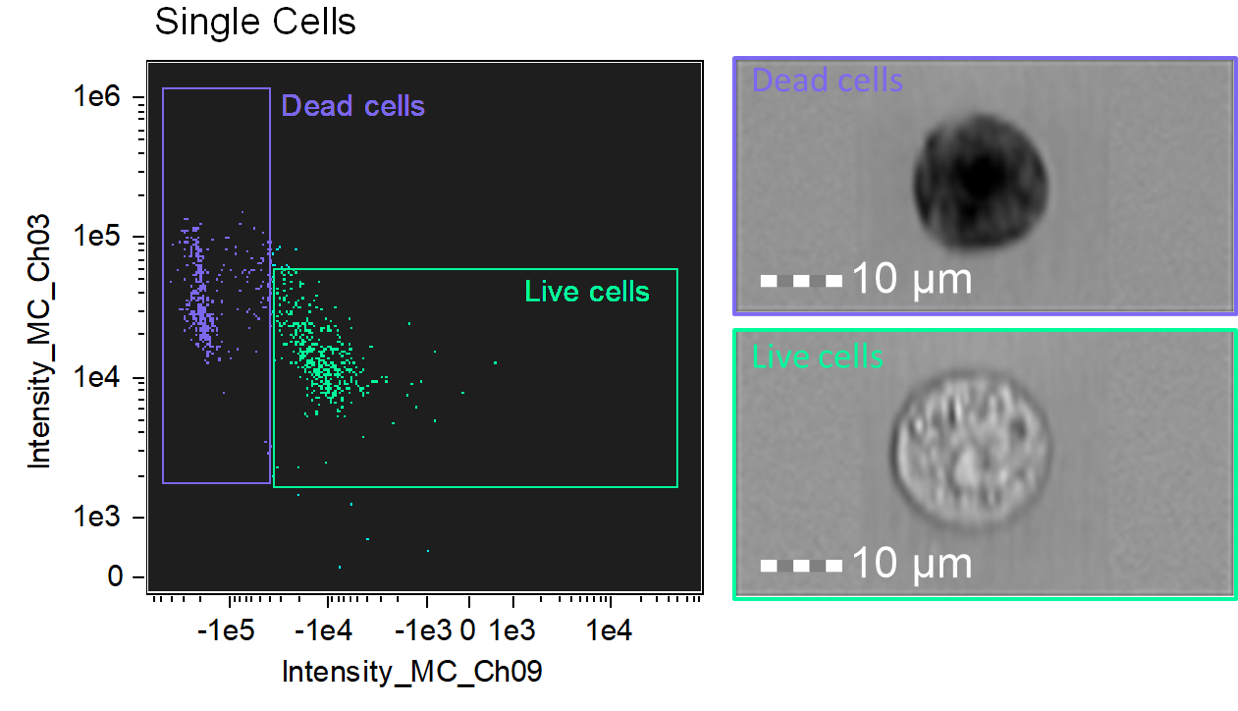


**Fig. S12**. Distinction between trypan blue stained and unstained cells

When particles are applied to the cells, a third population that contains live cells that have internalised particles is present (Figure S13A). This population contains cells, which have internal particles (Figure S13B) and cells that have both internal and external particles, however external particles are no longer fluorescent (F

igure S13C). Cells that have external particles and no internal particles are present in the live cells only region (Figure S13D) as the fluorescence from their particles has been quenched. Eq. S1 calculated the percentage of cells with internal particles.


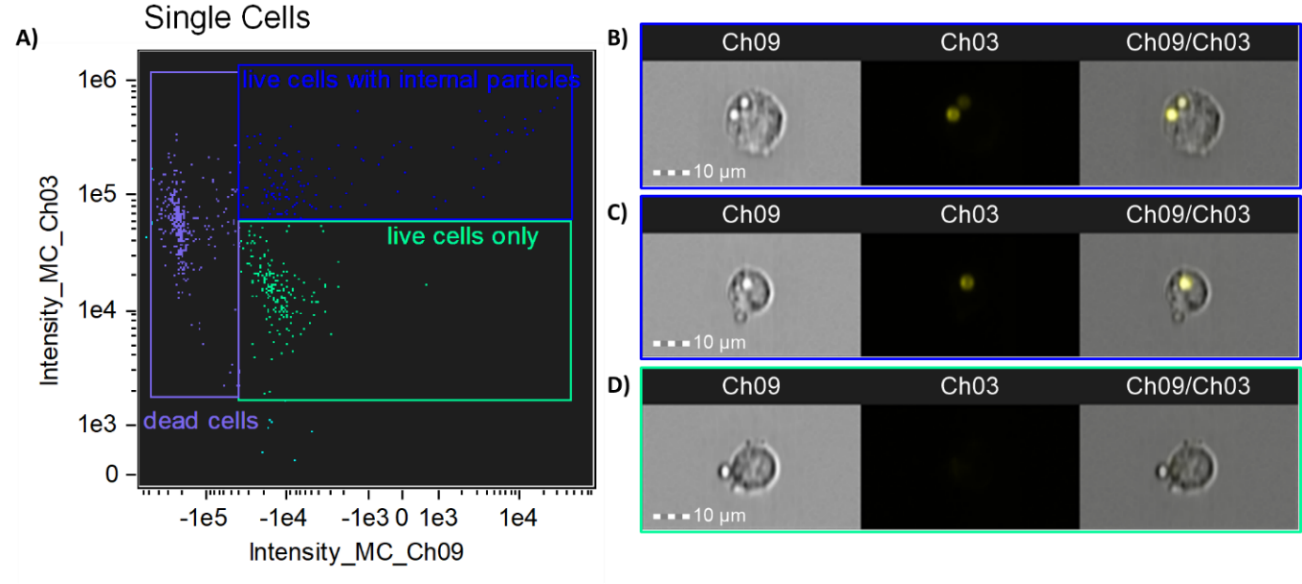


**Fig. S13**. Distinction between cells with surface-bound and internalised particles using the trypan blue quenching method. A) B) Brightfield and fluorescence images of a live cell with internal particles only C) Brightfield and fluorescence images of a live cell with both an internal and external particle D) Brightfield and fluorescence images of a live cell with a quenched surface-bound particle

Eq. (S1)

$$\boldsymbol{Cells with internal particles\% =}\frac{\boldsymbol{Number of live cells with internal particles}}{\boldsymbol{Number of live cells with and without internal particles}}\boldsymbol{\times100}$$

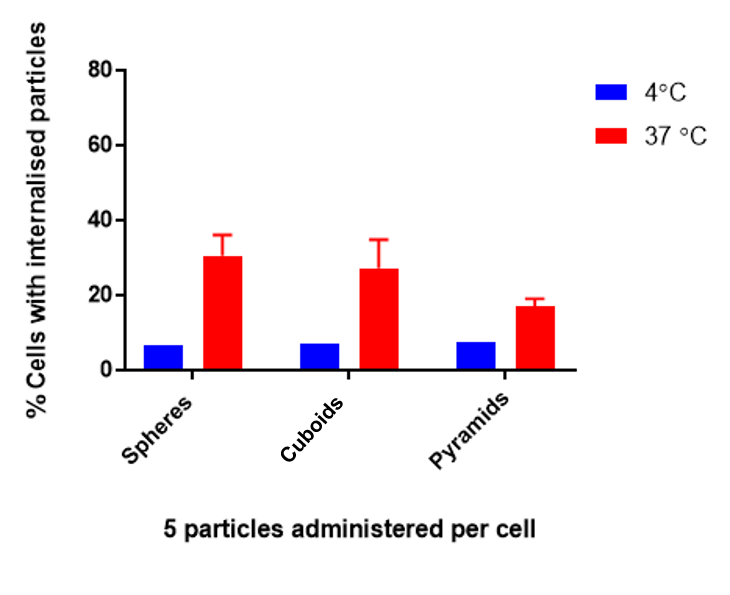


**Fig. S14**. Effect of temperature on phagocytosis. Cells were incubated at 4 °C for 30 minutes before administration of pre-chilled particles and for the duration of the experiment (4 hours). Cellular uptake was determined by IFC.


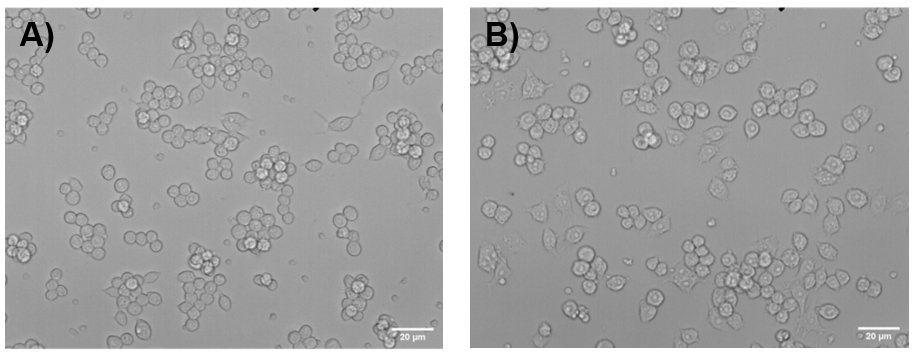


**Fig. S15**. Light microscopy images of RAW 264.7 cells incubated for 24 hours in A) Culture media, B) Culture media containing LPS


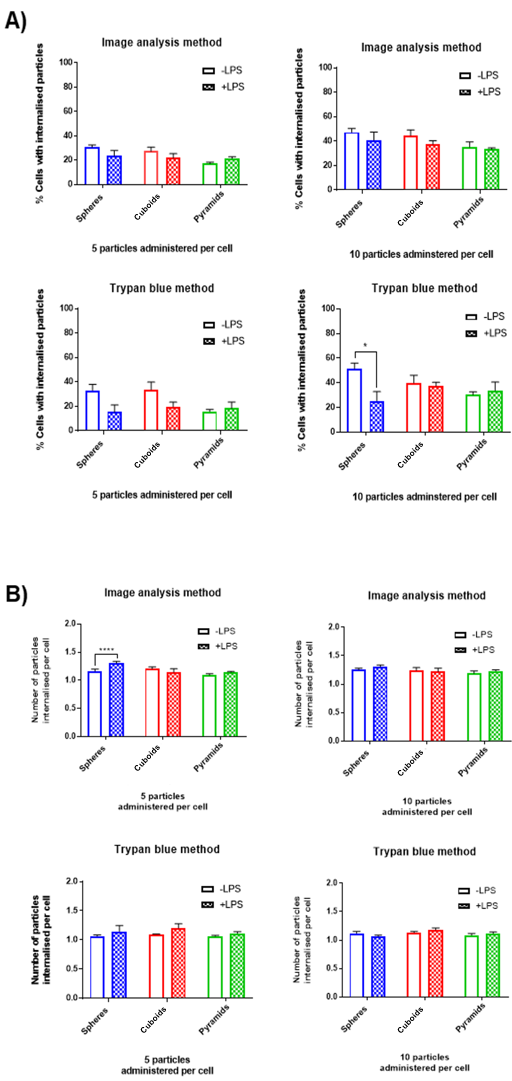


**Fig. S16**. Effect of LPS stimulation on particle uptake. The effect of LPS stimulation on A) % cells with internalised particles B) The number of particles internalised per cell. *, **, ***, **** indicate statistical significance (p < 0.05, 0.01, 0.001, 0.0001 respectively) as calculated by multiple unpaired T-tests. Values are representative of three biological repeats ± SEM (N=3).


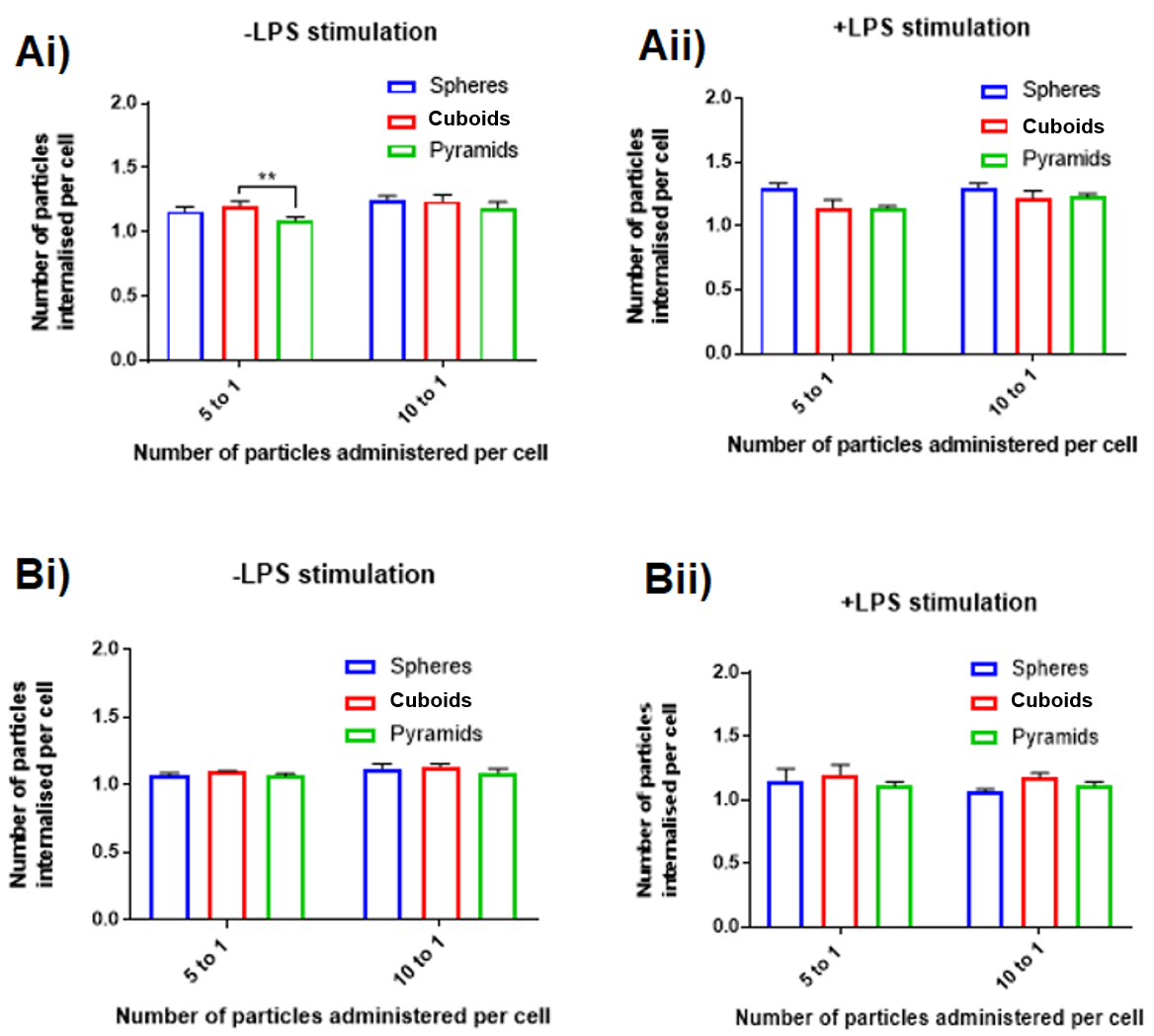


**Fig. S17**. The effect of particle shape on cellular uptake. The effect of particle shape on number of particles internalised per cell. Ai/ii) analysed using IFC, Bi/ii) analysed using TBQ method. *, **, ***, **** indicate statistical significance (p < 0.05, 0.01, 0.001, 0.0001 respectively) as calculated by 2way ANOVA with multiple comparisons. Values are representative of three biological repeats ± SEM (n=3).
